# Discovery of Octahydroisoindolone as a Scaffold for the Selective Inhibition of Chitinase B1 from *Aspergillus fumigatus*: In Silico Drug Design Studies

**DOI:** 10.3390/molecules26247606

**Published:** 2021-12-15

**Authors:** Alberto Marbán-González, Armando Hernández-Mendoza, Mario Ordóñez, Rodrigo Said Razo-Hernández, José Luis Viveros-Ceballos

**Affiliations:** 1 Centro de Investigaciones Químicas, Instituto de Investigación en Ciencias Básicas y Aplicadas, Universidad Autónoma del Estado de Morelos, Av. Universidad 1001, Cuernavaca 62209, Mexico; alberto.marbangon@uaem.edu.mx (A.M.-G.); palacios@uaem.mx (M.O.); 2 Centro de Investigación en Dinámica Celular, Instituto de Investigación en Ciencias Básicas y Aplicadas, Universidad Autónoma del Estado de Morelos, Av. Universidad 1001, Cuernavaca 62209, Mexico; ahm@uaem.mx

**Keywords:** bioinformatic analysis, chitinase AfChiB1, molecular docking, molecular similarity, oxazolinium ion

## Abstract

Chitinases represent an alternative therapeutic target for opportunistic invasive mycosis since they are necessary for fungal cell wall remodeling. This study presents the design of new chitinase inhibitors from a known hydrolysis intermediate. Firstly, a bioinformatic analysis of *Aspergillus fumigatus* chitinase B1 (AfChiB1) and chitotriosidase (CHIT1) by length and conservation was done to obtain consensus sequences, and molecular homology models of fungi and human chitinases were built to determine their structural differences. We explored the octahydroisoindolone scaffold as a potential new antifungal series by means of its structural and electronic features. Therefore, we evaluated several synthesis-safe octahydroisoindolone derivatives by molecular docking and evaluated their AfChiB1 interaction profile. Additionally, compounds with the best interaction profile (**1**–**5**) were docked within the CHIT1 catalytic site to evaluate their selectivity over AfChiB1. Furthermore, we considered the interaction energy (MolDock score) and a lipophilic parameter (aLogP) for the selection of the best candidates. Based on these descriptors, we constructed a mathematical model for the IC_50_ prediction of our candidates (60–200 μM), using experimental known inhibitors of AfChiB1. As a final step, ADME characteristics were obtained for all the candidates, showing that **5** is our best designed hit, which possesses the best pharmacodynamic and pharmacokinetic character.

## 1. Introduction

Invasive fungal diseases are a significant cause of morbidity and mortality in the growing population of immunosuppressed patients, such as transplant recipients, patients with hematological malignancies, and more recently, patients with severe COVID-19 [1]. In this high-risk population, the opportunistic pathogens of *Aspergillus* genera have emerged as the most frequent cause of invasive fungal diseases [2,3]. Although triazole drugs are commonly used as the first line of clinical therapy for invasive aspergillosis [4], numerous adverse clinical effects, such as neurotoxicity, nausea, vomiting and kidney damage, have been reported. Triazoles are inhibitors of the cytochrome P450 14α-sterol demethylase (CYP51), an essential enzyme in ergosterol biosynthesis in fungi. Still, they also interfere with the human CYP3A4 enzyme, increasing toxicity and the risk of drug-drug interactions in immunocompromised patients [5,6]. In addition, the occurrence of drug resistance in fungal pathogens is still a significant clinical problem, and ubiquitous fungi like *Aspergillus fumigatus* have exhibited drug resistance due to agrochemicals [7,8].

Hence, attention has turned to the discovery of new antifungal agents and finding other possible therapeutic targets. In this context, glycosyl hydrolase family 18 (GH 18) chitinases are enzymes that hydrolyze *N*-acetyl-β-D-glucosaminide (1–4)-β-linkages, of the highly abundant biopolymer chitin, interfering in fungal cell wall morphogenesis, remodeling and dynamic rigidity [9]. For example, the pseudotrisaccharide allosamidin is a transition-state mimetic and a selective inhibitor of family 18 [10,11] that inhibits cell separation in growing yeast of *C. albicans*, giving rise to clumps of cells [12], while its derivative, demethylallosamidin (DMT), causes an abnormal cell cluster formation affecting the growth of *S. cerevisiae* [13]. However, despite the potency of allosamidin, it is not commercially available and its synthesis remains complex and challenging [14]. Further evidence points out that some other chitinase inhibitors can also affect the hyphae morphology drastically in *A. fumigatus*, having a significant protective effect in invasive pulmonary aspergillosis [15], and also several inhibitors have been reported against chitinases from *A. fumigatus* [16,17,18,19,20] and *C. albicans* [21,22,23].

Unfortunately, few inhibitors exhibit good “hit-” or “lead-like” features. Recently, a virtual screening identified bioisosteric inhibitors with similar shape and electronic properties to the chitinase substrate, β-1,4-linked *N*-acetylglucosamine. Employing structure-based virtual screening combined with bioactivity assays, two new scaffold series were discovered—thienopyridine and furoquinoline—which exhibited inhibitory activity toward AfChiB1 at the micromolar range [24]. On the other hand, a virtual screening-based approach identified a novel purine-based AfChiB1 inhibitor, acting in the low micromolar range in a competitive mode. X-ray structural studies revealed that ligand stabilization relies on extensive π-π stacking interactions with conserved, solvent-exposed tryptophan’s (Trp137, Trp384, and Trp52). Thus, compound C2-dicaffeine exhibited favorable chemical properties and proved to be a valuable scaffold for the design of family 18 chitinase inhibitors [17]. In addition, ligands consisting of two linked caffeine moieties with a different number of methylene groups in the linker connecting the two xanthine ring systems showed IC_50_ values lesser than 10 μM for AfChiB1 and were selective compared to other human chitinases [18]. Moreover, the fungal natural product argifin is a broad-spectrum inhibitor of several family 18 chitinases [25]. Additionally, a structure-based dissection study identified dimethylguanylurea (DGU) as the minimal fragment of argifin necessary for competitive inhibition of a bacterial type chitinase through hydrogen bond formation, with the highly conserved residues Asp175, Glu177, Tyr178, Asp246 and Tyr245. Indeed, all the DGU-containing peptides showed IC_50_ values in the micromolar range [26].

This study presents the hydrolysis intermediate and AfChiB1 structure-based drug design of potential inhibitors, employing molecular docking—in a well-conserved crystal binding site and physicochemical properties of solvation—for selection of the best ligand, through energy stability in the binding site and selectivity over CHIT1. Thus, this scaffold proposal could serve as a basis for the future modification of the octahydroisoindolone bicyclic core to make promising chitinase inhibitors.

## 2. Computational Details

### 2.1. Bioinformatic Analysis

**Figure 1:**
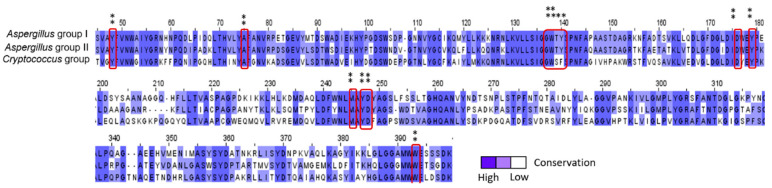
Fungi consensus sequences and highlighted main amino acid residues in the binding site. *Aspergillus* consensus sequence group I and II represents various *Aspergillus* species, and the *Cryptococcus* group is composed of *neoformans* variant *grubii* and *gattii* species. * Refers to low conservated sequences and $\begin{array}{c} {}*\\ {}* \end{array}$ refers to high conservation in sequences.

The protein sequence was chosen from the crystal structure of the AfChiB1-inhibitor complex [24]. We used this amino acid sequence to search in the Protein-NCBI webserver for similar length sequences and retrieved 4254 results. JalView 2.11.1.3 software [27] was used for multiple sequence alignment and clustering. All the sequences of chitinases were clustered in two *Aspergillus* genera and one *Cryptococcus* variant, with 22 and 7 sequences respectively (Figure S1, see Supplementary Data). As described in Figure 1, the active site is highly conserved for all consensus sequences, except for the *Cryptococcus* group. Then, we submitted each fungi consensus sequence in the Protein-NCBI webserver for *Homo sapiens* searching; these afforded three clusters, generating three human consensus sequences.

### 2.2. Homology Structure Modeling

We generated all structures of the found consensus sequences to contrast between fungi and human chitinases. To resolve this task, we employed the I-TASSER server; according to CASP competitions, this webserver makes accurate predictions for structure protein modeling based on a template by sequence similarity [28,29]. Summarizing the model results, we found acceptable values that the server utilizes to validate protein structure. The normalized Z-score (Z-score) indicated good alignments with PDB library, the C-score (range of −5 to 2) had higher values in the range for the quality of predicted models, and the TM-score indicated a good structural similarity to different chitinases organisms. Templates used by I-TASSER for consensus sequence-structure assembly of *Aspergillus* group I were based on chitinases from *A. fumigatus*, *C. immitis*, *C. rosea* and *Y. entomophaga*. The coverage of the alignment of the threading template was more than 0.96 and the percentage sequence identity was around 0.93 for *A. fumigatus*, while the Z-score was greater than one. In addition, identity by TM-alignment showed structure similarity to different chitinases species (TM-score > 0.9). Similarly, for the homology model construction of consensus sequences of *Aspergillus* group II and the *Cryptococcus* group, chitinases were employed as templates. On the other hand, the coverage of the threading alignment (>0.87), percentage sequence identity (0.51 to 0.63), Z-score (>1.0) and TM-score (>0.77) were suitable for template assembly.

I-TASSER constructed *Homo sapiens* proteins mostly from chitinases with values of threading alignment (>0.91), percentage sequence identity (from 0.4 to 1.0 for the three sequences), Z-score (>1.28) and TM-score (>0.85). As a result, amino acid residues in the binding site showed differences with the examined fungi chitinases.

### 2.3. Scaffold Proposal

Scaffolds were designed based on the oxazolinium ion formed during chitin hydrolysis; its known analog is the allosamidin that contains an oxazoline ring. Thus, geometry and the bioisosteric replacement approach were used for the construction of the intermediate fragment. Our proposals consisted of a cyclohexane ring [*c*]-fused to a γ-lactam, giving ten possible structures, which were submitted to a conformer analysis calculation, using the semiempirical PM6 approximation [30], generating four bowl-like conformers with similar dihedral angles for the reaction intermediate. Hence, four scaffolds were selected according to the dihedral angles of the reaction intermediate. Additionally, a single-point calculation using the density functional theory approach [31] with the functional and basis set M06/6-31 + G* [32] was employed. SPARTAN’18 was used for calculations [33].

### 2.4. Molecular Docking Calculations

All the ligands used for these calculations were constructed and geometrically optimized using semiempirical AM1 theory. Two partial charges schemes were evaluated: electrostatic and Mulliken. All these calculations were performed with SPARTAN’18.

**Figure 2:**
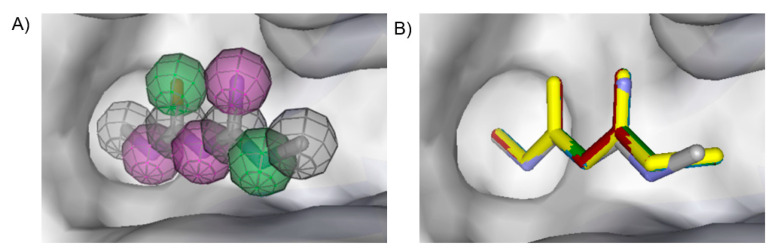
(**A**) Ligand template set up for docking; spheres indicate hydrogen acceptor (green), hydrogen donors (purple) and steric (gray). (**B**) Dimethylguanylurea (DGU) conformer reproducibility of four independent runs in the AfChiB1 active site.

We used the crystal structure of AfChiB1 from the protein data bank (PDB) with code 3CH9, and a 2.20 Å resolution. All water molecules were removed, and structure details were corrected. The co-crystallized ligand of 3CH9 was set up as a template for molecular docking (atoms were specified as in Figure 2), considering the amide group orientated to (α/β)8-barrel fold in the binding site (volume cavity is 386.11 Å^3^). Different scoring functions and search algorithm parameters (such as the number of runs, maximum iterations, and population size) were tried, to reproduce the structural conformation of the ligand in the cocrystal complex. Finally, the ligand electrostatic partial charges, as well as the MolDock score (GRID) scoring function (with 0.2 Å (grid resolution), 12 Å radius of search sphere) and the MolDock optimizer search algorithm (with 20 runs and a maximum of 4000 iterations for a population size of 200 individuals) were the best to reproduce DGU’s conformation. The docking process was validated with conformer reproducibility of DGU with a root mean square deviation (RMSD) less than 1 Å, and the poses selected according to orientation at the cavity, as described in Figure 2. Molegro Virtual Docker (MVD) 6.0 was used to perform all the docking calculations [34].

### 2.5. Construction of Ligands Derivatives

On the other side, to discriminate scaffold proposals, poses were inspected visually by considering orientation, and intramolecular and intermolecular clashes. Several modifications of the four scaffold proposals (easily accessible through organic synthesis, mainly C-3 substituted derivatives) gave 76 structures. As a result, from these rigid docking calculations, we identified a preferred scaffold stereochemical configuration in the AfChiB1 complex. Next, we designed new C-3 substituents with the aliphatic chain (CH_2_)_2_R and its homolog structure (CH_2_)_3_R, where R represents a heterocyclic or heteroaromatic fragment, giving 38 derivatives. Partition coefficient octanol-water (aLogP) was also employed to select the best ligands in correlation with their interaction energy [35]. To contrast chitinase selectivity, the best ligands were evaluated in human chitotriosidase-1 (CHIT1, PDB: 5NR8) using the same rigid docking method as described above. This chitinase is relevant because it is produced mainly by macrophages and could play a role in defense against fungal infections [36,37].

### 2.6. Re-Docking and Selectivity Analysis over CHIT1

The best poses from our previous analysis were submitted to a flexible docking calculation by setting a template based on the best results obtained in the first docking. Then, amino acid residues were selected to be flexible and parameters such as tolerance were set to 0.9 for all residues and strength was applied as described in the Supplementary Data (Table S29). The strength parameter points out that zero values are set to very flexible sidechains [38]. This approach was extended to CHIT1, then the resulting interaction energies and cavities were analyzed. Additionally, the aLogP value was determined for all candidates.

### 2.7. Mathematical Model for IC_50_ Prediction

The same approach was applied to AfChiB1 inhibitors (caffeine, dicaffeine, PTX, DGU, TPH, **6**, **7** and **8**), and their MolDock score and aLogP [39] values were used for the construction of a mathematical model for the IC_50_ prediction. Therefore, these values were considered as independent variables, and experimental IC_50_ values as the dependent variables. Nevertheless, to generate a quality model, a logarithmic transformation over IC_50_ was done; this model was constructed with Excel Microsoft Office 365. Standard deviation (s), Fishers F-test (F), and the coefficient of determination ($R^{2}$) were used for the model validation. For the predictive ability evaluation of the model, we used the cross-validation coefficient ($Q_{\mathrm{LOO}}^{2}$) with the Leave-One-Out (LOO) method; due to the small number of compounds used for this model, $Q_{\mathrm{LOO}}^{2}$ gives us a quality evaluation of its prediction ability [40].

## 3. Results and Discussion

### 3.1. 3D Structure Protein Analysis

**Figure 3:**
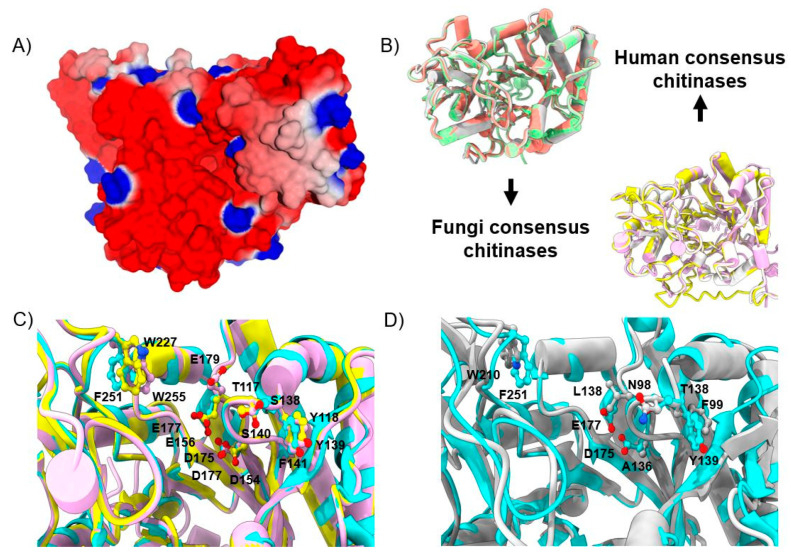
AfChiB1 crystal structure protein data bank with code 3CH9 (PDB: 3CH9) and structural alignment with consensus homology models. (**A**) Molecular electrostatic potential (MEP) surface and catalytic cavity (highlighted by a rectangular form) of chitinase AfChiB1; blue, red and white colored regions indicate positive, negative and zero values of MEP, respectively. (**B**) Structural alignment of the fungi consensus model, and human consensus chitinases. (**C**) Catalytic site of fungi-predicted structures; 3CH9 representation is colored in blue. (**D**) Structural alignment of human consensus model against 3CH9 colored in blue. Key residue differences are highlighted by ball and stick representation.

We constructed chitinase 3D homology models for each consensus sequence, with good I-TASSER C-scores, within positive values indicating good quality. C-score values for the *Cryptococcus* group, *Aspergillus* group I, and *Aspergillus* group II were 0.16, 1.5, and 0.98, respectively. The three structures were aligned with the crystal structure of AfChiB1 (RMSD less than 1 Å). The catalytic site is very conserved and is exposed to the solvent. According to its molecular electrostatic potential (MEP) surface, most of the cavity display a negative MEP value, and only a small region displays a positive MEP value, where Arg57 and Arg301 are located (Figure 3A). After the structural alignment of the *Cryptococcus* group, there were two residue differences detected: Thr138 changed to Ser140 and Tyr139 to Phe141 (Figure 3C); aside from these, there were no significant changes in amino acids between the two groups.

Figure 3D shows the structural alignment of human consensus chitinases with AfChiB1 (3CH9), and some differences in amino acid residues close to the binding site, heightening mutants in AfChiB1 and the three human consensus structures: Thr138 (all sequences had Asn), Tyr139 (all sequences had Phe), Asp175 (only Ala 136 in human sequence from *Aspergillus* group II), Glu177 (Leu 138 from *Aspergillus* group II) and Phe251 (all sequences had Trp).

### 3.2. Scaffold Design

**Figure 4:**
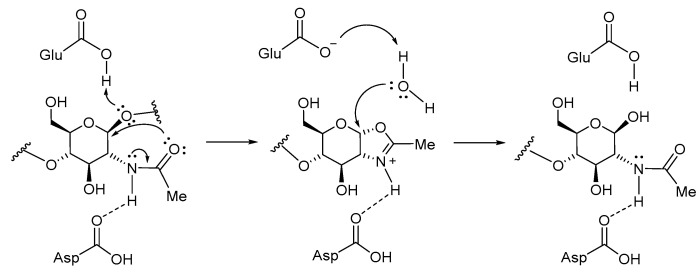
Hydrolysis mechanism for GH 18 chitinases.

**Figure 5:**
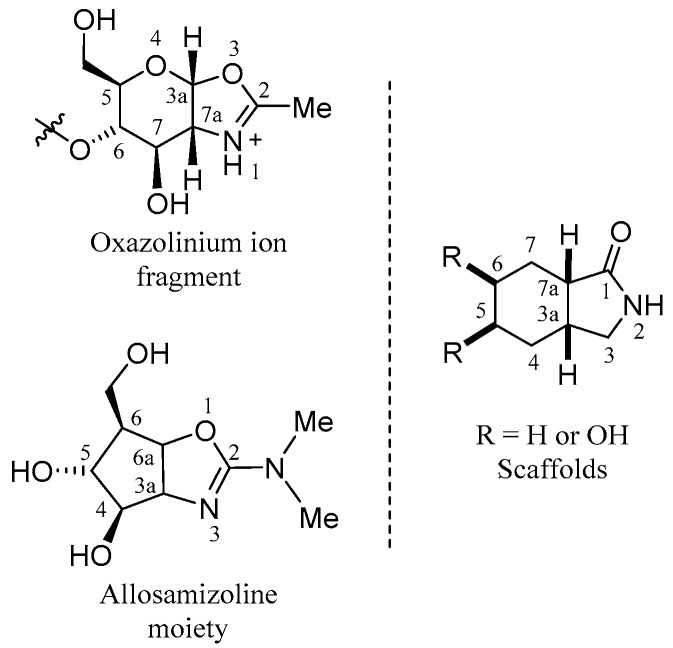
Reaction intermediate, allosamizoline crystal structure, and scaffold structures.

We analyzed the reaction intermediate proposed during the hydrolysis mechanism in the GH 18 family, given by an acetamide intramolecular nucleophilic attack at the C-3a position (glycosidic bond carbon) that generates an intermediate oxazolinium ion, giving rise to a constrained pyranose ring fused to an oxazole ring (Figure 4). This skeleton was maintained with cyclohexane and a five-member ring lactam. The bioisosteric replacement to an amide group was thought to electronically mimic the oxazolinium ion formed due to glycosidic bond rupture. Then, some scaffolds could have hydroxyl groups in carbon C-5 and C-6 to maintain a glycosidic-like structure (Figure 5).

**Figure 6:**
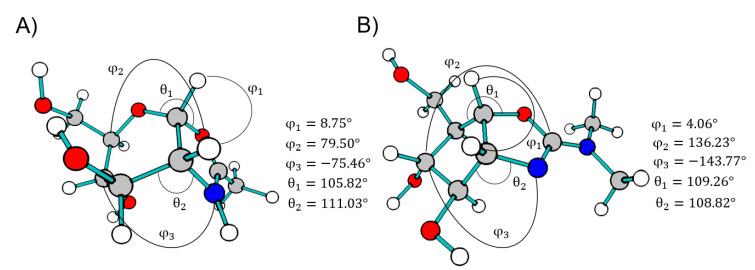
Values for dihedral angles: φ_1_ (H-3a-7a-H), φ_2_ (C5-O4-C3a-O3) and φ_3_ (C6-C7-C7a-N1) for oxazolinium ion minimum energy (**A**), and φ_1_ (H-6a-3a-H), φ_2_ (C5-C6-C6a-O1) and φ_3_ (C5-C4-C3a-N3) for allosamizoline crystal moiety (**B**, PDB: 2A3E) and θ fused ring angles.

We performed a conformer analysis of this hydrolysis intermediate, using PM6 approximation, obtaining 19 conformers. From these geometries, the angles were analyzed and considered for the ligand-based design, and the minimal energy conformation was selected which had a geometry-like bowl (Figure 6 and Table S25, see Supplementary Data). Besides, electronic properties were examined by single-point energy calculations of each conformer with DFT, used to explore frontier molecular orbitals and molecular electrostatic potential of organic compounds [41,42,43,44,45]. Thus, we used the allosamizoline ligand (PDB: 2A3E) as a reference because it shares structural characteristics with the oxazolinium ion intermediate (Figure 5 and Figure 6) [46]. We found the molecular geometry of the oxazolinium ion in its minimum energy conformation with dihedral angles of φ_1_ = 8.75°, θ_1_ = 105.82° (O4-C3a-O3) and θ_2_ = 110.03° (C7-C7a-N1), which were similar to the allosamizoline conformation.

**Figure 7:**
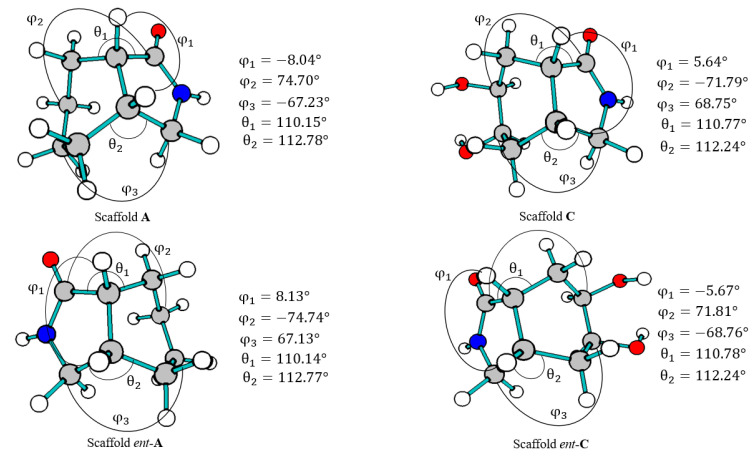
Values for dihedral angles: φ_1_ (H-3a-7a-H), φ_2_ (C6-C7-C7a-C1) and φ_3_ (C5-C4-C3a-C3) and θ fused ring angles of each scaffold conformer, similar to the allosamizoline moiety and the oxazolinium ion fragment.

On the other side, scaffold design was performed on the basis of reliable synthetic methods, like those described in Meyers’s lactams synthesis for the preparation of *cis*- and *trans*-fused lactams [47,48,49]. As a result, from conformational analysis in comparison to oxazolinium ion and allosamizoline moiety, only in Figure 7, the *cis*-fused scaffolds and their enantiomers had similar angles (Table S26, see Supplementary Data). Geometry gets closer to the oxazolinium ion because scaffolds and intermediates had six-five fused rings and allosamizoline had a five-five fused system. Further, φ_1_, θ_1_, and θ_2_ angles indicate similar amide orientation in scaffolds, as oxazole shows in the structure references.

Additionally, electronic properties were explored. We found similarity of the oxazolinium ion by the lowest molecular orbital (LUMO) region located in oxazole ring, same region for the electron acceptor site. This agreedwith the chitinase GH 18 hydrolysis mechanism, that involves an intramolecular nucleophilic attack by the oxygen of the acetamide to the anomeric carbon (Figure 4) [50,51,52,53].

**Figure 8:**
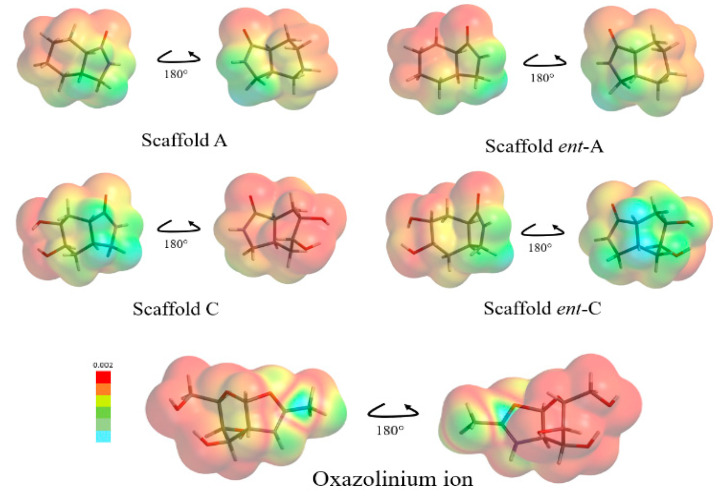
Lowest molecular orbital (LUMO) map of scaffo ld proposals in comparison with the oxazolinium ion fragment. Colors go from red (minimum value) to blue (maximum value) according to the absolute value of the LUMO on the electron density surface.

Moreover, the scaffold proposals had a similar LUMO map in Figure 8, depicted by the amide group that mimics the oxazole ring electronically, forming the electrophilic region on the five-membered ring-like intermediate.

**Figure 9:**
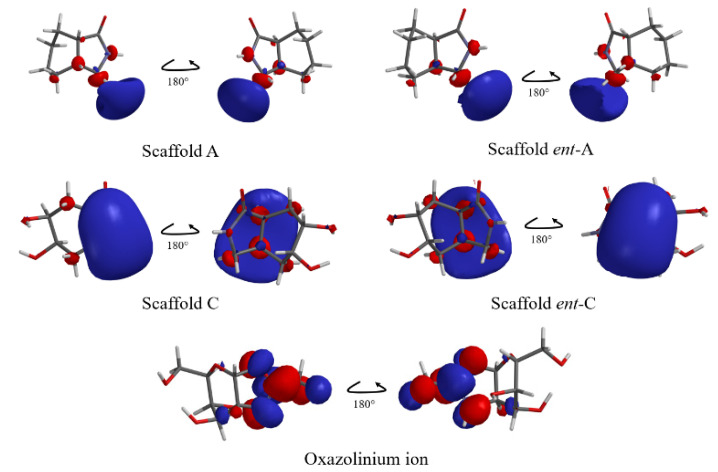
Lowest Unoccupied Molecular Orbital graphic of the scaffold pro posals and the oxazolinium ion. Shape and size of the graphics shows the contribution to the LUMO of each atom in the molecule.

This electronic similarities are more evident when LUMO orbital graphics are displayed, as illustrated in Figure 9. LUMO graphics are located in the five membered ring.

On the other hand, scaffolds and oxazole rings have three types of atoms that showed correlated Mulliken partial charges; carbon (sp^2^ hybridization) had positive charges, thereby nitrogen and oxygen had negative charges for all scaffolds and the oxazolinium ion (Table S27, see Supplementary Data). In summary, the scaffold conformers presented similar geometry to the oxazolinium ion. Some electronic parameters correlate our proposals with the reaction intermediate, suggesting similar interactions on the atoms in the five-membered rings. Thus, the next step was to seek scaffold derivatives through a structure-based design that relied on docking calculations.

### 3.3. Structure-Based Drug Design

**Figure 10:**
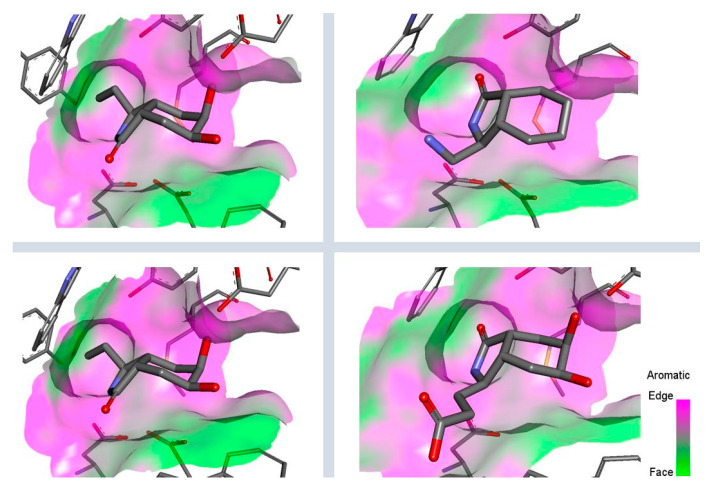
Scaffold modifications **A**, *ent*-**A**, **C**, and *ent*-**C** in the C-3 position for group I and II. Surface plot of the catalytic site, showing the aromatic interaction profile. Group I (R = −(CH_2_)n − Z, n = 1 to 3, Z = functional group) and group I (R = −(CH_2_)n-CH_3_, n = 1 to 6). The face label indicates a face interaction between amino acid residues and ligands’ hydrophobic fragments, and the edge label shows the noncoplanar amino acid residues orientation to the protein surface.

For this set analysis we employed a rigid docking approach; hence, Figure 10 shows the scaffold derivatives design that consisted of the substitution in the C-3 position, because the access to enantiomerically pure compounds was rationalized by a nucleophilic attack via the acyliminium ion as a reaction intermediate furnishing the substituted lactams [48,54]. The first design step explored the functional groups effect by placing hydrocarbon chains by one to three carbons, and bound to functional groups like amines, thiols, alcohols, or carboxylic acids (group I). On the other hand, the hydrophobic substituents were set by one to six carbons homologation to seek possible clashes or to increase chain stability through interactions with aromatic residues like Trp137, Tyr48, and Trp384 in the active site (group II).

Ligands were discriminated according to their interaction energies and selected by visual inspection of their binding modes [55], as well by the ligand orientation of the bioisosteric group towards the (α/β)_8_ barrel fold. As mentioned above, *ent*-**C** derivatives from group I in Figure 10 had a slight preference by AfChiB1 interacting mainly with Tyr48, Gly136, Trp137, Asp175, Glu177, Asp246, Tyr245, Met243 and Trp384. Hence, most stable poses were derived from scaffolds with primary amines, followed by thiol and alcohol chains; this suggests that protonation at the physiological pH of amines provides stability. In addition, *ent*-**C** substituted carboxylates showed more stability by hydrophobic interaction enhancement between Trp384 and the scaffold lactam ring, than the previously mentioned derivatives. Additionally, in Figure 10, the scaffold modification of *ent*-**C** presented a noticeable preference of AfChiB1 in group II, since they showed better interaction through Trp384 and Trp137 stabilization, and hydrogen bonds with Glu177 and Tyr245.

**Figure 11:**
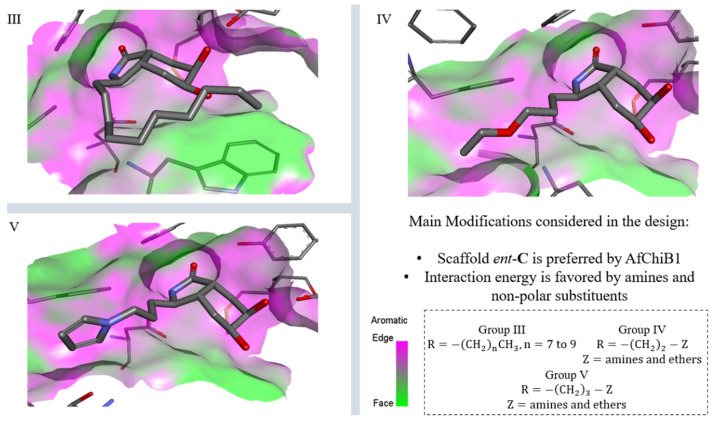
Scaffold *ent*-**C** modifications in the C-3 position for groups III, IV and V. Surface plot of the catalytic site, showing the aromatic interaction. The face label indicates a face interaction between amino acid residues and ligands’ hydrophobic fragments, and the edge label shows the noncoplanar amino acid residues orientation to the protein surface.

From this screening, we identified a stereochemical preference of AfChiB1 and promising auxophoric groups like amines, ethers, and non-polar chains, by constructing group III to V of derivatives based on scaffold *ent*-**C**. In Figure 11, the derivatives in group III were designed to elongate the hydrocarbon chain to 7–10 carbons; nevertheless, they showed similar affinity energies to group II.

Furthermore, structures of Figure 11 showed groups IV and V with amine substituents generally with better energies than groups I–III. Then, derivatives with amines directly bonded to aromatic or aliphatic rings presented the lowest energy, predominantly through stronger interactions by Phe76, Trp137, Thr138, Tyr 139, Glu177, Tyr245 and Trp384.

**Figure 12:**
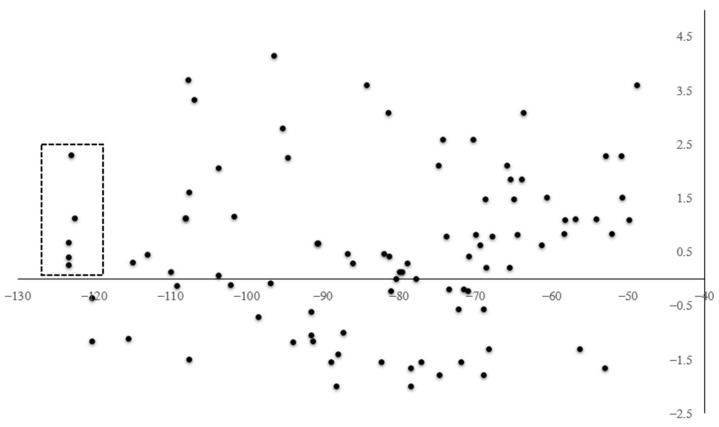
Moldock score (kcal/mol, x-axis) vs. aLogP (y-axis). The marked region indicates the final candidates.

Then, all proposed derivatives constituted a large set to discriminate based on their interaction energy, without considering the pharmacokinetic criteria. Therefore, aLogP was considered for the selection of the final candidates, taking the best value range between 0 and 3 for acceptable drug absorption, as was highlighted in Figure 12, identifying five structures as the best candidates.

### 3.4. Selectivity Analysis over AfChiB1 and CHIT1

Each pose from the previously selected five structures was submitted to a second docking calculation and examined, identifying hydrogen bonds and other important non-covalent interactions (Table 1). In this way, **1** had interactions with Trp384, Asp246, Glu177, Trp137, Thr138, Tyr 139, Gly136, Phe76, Tyr245, Met243, Tyr48, and Asp175. Ligand **1** showed a hydrogen bond from amide NH and the carboxylate Asp175. Furthermore, amide carbonyl interacts through hydrogen bonding to the hydroxyl Tyr245, and hydroxyl in the C-5 position of **1** forms a hydrogen bond to Asp246. The phenyl ring A was orientated towards Trp137 and exposed to the solvent. On the other hand, aromatic ring B was orientated to Phe76, Trp52, Gly136, Thr138, and Tyr139 residues. Ligand **2** had a phenyl ring proximity identical to aromatic non-polar residues as the orientation of ring B in pose **1** and hydrogen bonds with Asp175 and Tyr245, besides C-5 and C-6 hydroxyl groups interacted with Glu177 and Asp246, respectively. On the other side, **3** only presented hydrogen bonding to Tyr245, Asp246 and Glu177, but the phenyl was exposed to solvent and orientated to Trp52 and Trp384. Thus, **4** kept the hydrogen bond to Tyr245 and hydroxyls formed two hydrogen bonds with Asp246, and the cyclohexyl ring was orientated, as **1** and **2** did. Finally, **5** presented a similar pose as **2**, keeping hydrogen bonding to Asp175, Glu177, Tyr245 and Asp246, as well as the phenyl orientation (Tables S30–S34, see Supplementary Data).

The next step was to evaluate the best ligands for CHIT1 by a rigid docking approach; however, pose orientation criteria was not found for **5**. Then, flexible docking calculations were performed for set **1**, **2**, **3**, and **4**, employing some flexible residues equivalent to the AfChiB1 sequence by structural alignment near the active site. Furthermore, from bioinformatic analysis, mutations between *A. fumigatus* and human were identified since there were null interactions in flexible docking for AfChiB1 with Phe251 and Tyr247. In contrast, **1**, **2**, **3** and **4** had non-polar stabilization interactions by residues Trp218 and Phe214, which are the analogue mutants’ residues for CHIT1 (PDB: 5NR8).

The main interactions in the docking calculations of all the tried ligands with CHIT1 were Asp213, Trp99, Trp458, Met210, Tyr141, Tyr267, Trp218, Tyr212, Ala186, Phe214, Glu140, Tyr190, Pro185 and Gly187. In Table 2, we inspected **1**, thereby amine presented a salt bridge with Tyr141, and carbonyl formed a hydrogen bond to Tyr212. Its phenyl ring A was positioned close to Gly187, Ala186, Tyr141, Phe214, and Tyr190, while phenyl ring B was near Trp218 and exposed to solvent. **2** presented a salt bridge with Tyr141, besides hydrogen bonding to Trp358 with the same substituent orientation. On the other side, **3** had the same carbonyl interactions as **1**, but hydroxyl C-5 made a hydrogen bond to Asp213, and the phenyl substituent had a similar orientation to the phenyl ring A. For the last ligand, **4**, Asp213 presented a salt bridge between amine and hydrogen bonding with hydroxyl C-5, and carbonyl kept the same hydrogen bond interaction, while cyclohexyl had a similar orientation to **1**, **2** and **3** (Tables S35–S38, see Supplementary Data).

On the other hand, *A. fumigatus* had the major interaction energy because of stronger interactions with Trp384, Trp137, Phe76, Thr138, Trp52, Tyr139 and Asp246 residues; **2**, **4** and **5** displayed similar complex energy but lesser that **1**, while **3** only had the main interactions with Trp384, Trp137, Glu177 and Tyr245. In comparison, **1** in CHIT1 showed slightly stronger interactions with Asp213, Trp99, Trp358, Tyr212, Met210, Tyr141, Tyr267 and Ala 186 residues, while **2**–**4** kept lesser strong interactions with Asp213, Trp99, Trp358, and Tyr212 (energy interaction values of the final candidates from flexible docking with AfChiB1 and chitotriosidase with aLogP values are displayed in Table S47, see Supplementary Data). However, we cannot correlate these designed ligands’ binding energies for the estimation of drug potency, like Glide or Vina can perform. Then, a predictive model was developed, using the experimental IC_50_ values of AfChiB1 inhibitors and their MolDock score and aLogP values.

### 3.5. Mathematical Model for the IC_50_ Prediction over AfChiB1

Flexible docking parameters were set up in an identical way for reported inhibitors (considering the ligand DGU as a template, like was established for the drug-based design in AfChiB1), interactions were reviewed and verified for inhibitors with xanthine moieties (Tables S39–S48, see Supplementary Data). For example, available crystals and calculated poses kept most of interactions (Caffeine, PTX, DGU and TPH), while **6**, **7** and **8** were checked for amide orientation towards the active site.

**Figure 13:**
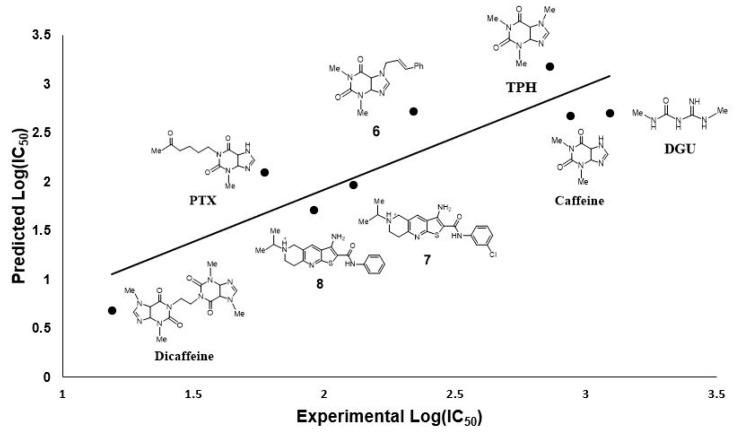
Inhibitors structures used for the model construction: caffeine (PDB: 2A3B), pentoxifylline (PTX; PDB: 2A3C), DGU (PDB: 3CH9), inhibitors **6** [15], **7** [22], **8** [22] and theophylline (TPH; PDB: 2A3A), and the linear relationship between ${\mathrm{IC}}_{50\mathrm{pred}}$ and ${\mathrm{IC}}_{50}$.

Then, using logarithm transformation over IC_50_ helped us to find a linear correlation with MolDockscore (interaction energy) and aLogP as independent variables, generating Equation (1). The linear correlation between ${\mathrm{IC}}_{50\mathrm{pred}}$ and ${\mathrm{IC}}_{50}$ is displayed in Figure 13. According to $Q_{\mathrm{LOO}}^{2}$, our model has a good predictive capability (${\mathrm{IC}}_{50\mathrm{pred}}$ values are shown in Table S48, see Supplementary Data). From Equation (1), the importance of the interaction energy with AfChiB1 is displayed; according to its coefficient, highly negative MolDockscore values (more stable complexes) will increase the potency of the inhibitors. In addition, the aLogP coefficient indicates that hydrophilic compounds are preferred to increase the potency of the compounds. Nevertheless, we need to consider the type of the biological test for the determination of the ${\mathrm{IC}}_{50}$ values, which do not consider a biological barrier. Therefore, we propose values of aLogP close to 1 for the design of this class of inhibitors. It is worth mentioning that the presence of these two variables in our model allowed us to study the inhibitors potency in two separated ways, its pharmacodynamic (MolDockscore), and the pharmacokinetic (aLogP) character.

(1) $$Log\left(IC_{50}\right)=0.01843MolDockscore+0.1609aLogP+4.3994$$

$$s=0.26\;\;\;\;\;\;\;F=29.11\;\;\;\;\;\;\;R^{2}=92.1\;\;\;\;\;\;\;Q_{LOO}^{2}=78.9\;\;\;\;\;\;\;\;n=8$$

Nevertheless, the model is limited to bicyclic derivatives, and rigid molecules, preferably. This assumption is based on the ability of our model to predict, in a better way, $IC_{50}$ values for caffeine, TPH, **7** and **8** (Figure 13, vide supra). Then, using Equation (1), we predicted the $IC_{50}$ values for our designed ligands in Table 1, estimating the concentrations of them to be around 60–200 μM for **1**–**5**.

For a complete drug design scheme, we determined the toxicity and drug-likeness of these candidates through the SwissADME webserver [56]. According to these calculations, final designed ligands will not inhibit cytochrome p450 isoforms and can be substrates for P-gp, a glycoprotein involved in pumping xenobiotics out [57]. Only **1** could permeate the blood–brain barrier (BBB), and it is predicted to be poorly soluble in water. Further, the expected bioavailability would be around 55% for all ligands; in general, they satisfied the main rules for drug design, like Veber, Lipinski, and Ghose.

In summary, docking in fungi and human chitinase suggests that **5** is a potential inhibitor of AfChiB1, since **5** could have a different orientation in the CHIT1 active site. At the same time, **1** will have the highest potency for AfChiB1 and CHIT1, and also possesses a good drug-likeness and pharmacokinetic profile, but the possibility of crossing the BBB will not be desirable for its biological application (Table S50, see Supplementary Data).

## 4. Conclusions

The bioinformatic analysis revealed that chitinases from different pathogenic *Aspergillus* taxa, including *A. fumigatus*, *A. novofumigatus*, *A. niger*, *A. candidus*, *A. fischeri*, *A. flavus*, and the *Cryptococcus* taxon of the *grubii* variant, are well conserved. A comparison of amino acid changes around the active site reported in human chitinases have also been documented. Eventually, we showed that the proposed scaffolds could mimic the oxazolinium ion concerning its conformation and electronic properties. On the other hand, we made a structure-based drug design, identifying the *ent*-**C** scaffold as preferred by the enzyme AfChiB1, allowing the selection of **1** to **5** ligands according to their aLogP values. In contrast, analysis with human chitotriosidase indicated that the most promising inhibitor is **5** for AfChiB1. The employed structure-based drug design and virtual screening protocol not only demonstrates its efficiency, but also provides novel and selective compounds for developing AfChiB1 inhibitors to protect against opportunistic invasive mycosis.

## Figures and Tables

**Table 1:** Best pose of the best scaffold derivatives (ligands) in AfChiB1.

Ligands	Pose in Cavity	Interactions
**1**	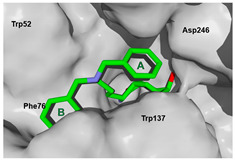	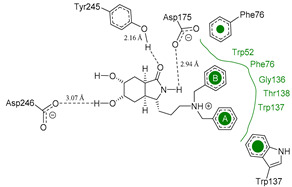
**2**	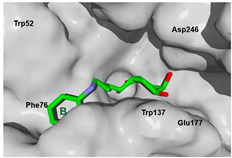	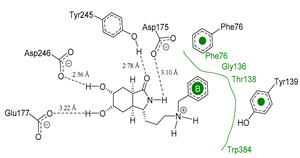
**3**	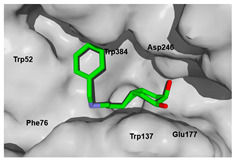	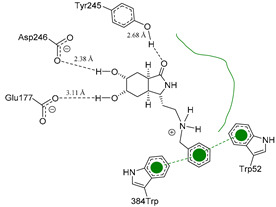
**4**	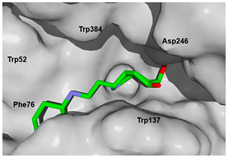	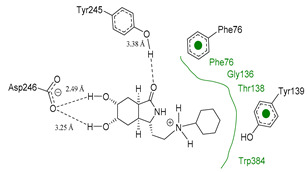
**5**	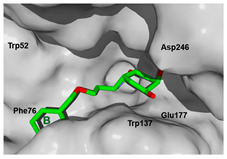	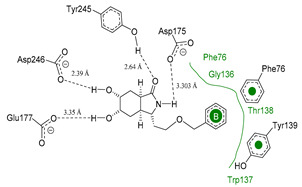

**Table 2:** Best pose of scaffold derivatives (ligands) in *Homo sapiens* CHIT1.

Ligand	Pose in Cavity	Interactions
**1**	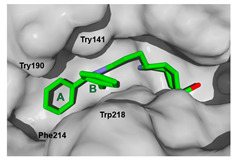	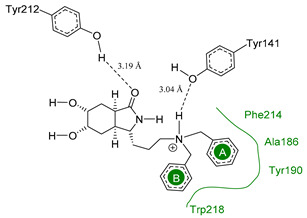
**2**	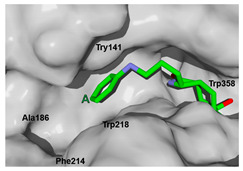	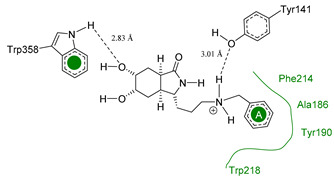
**3**	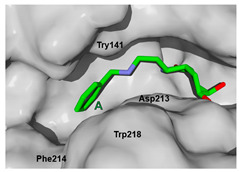	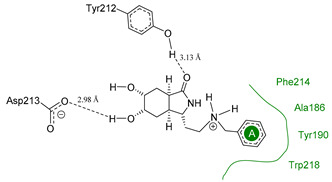
**4**	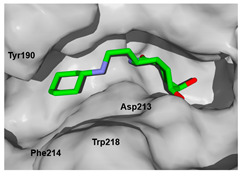	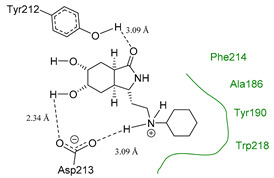

## Data Availability

The data presented in this study are openly available in PDB database, ID: 3CH9 and 5NR8.

## Supplementary Materials

The following are available online, Figure S1: Phylogenetic profile for fungi, Figure S2: Pose of each compound obtained from the re-docking analysis, Figure S3: Pose of each compound obtained from the re-docking analysis, Figure S4: Structures used for model construction, Table S1: Consensus sequence of *Aspergillus* genera (group I), Table S2: Top 10 threading templates used by I-TASSER, Table S3: Top 10 Identified structural analogs in PDB, Table S4: Best model predicted by I-TASSER, Table S5: Consensus sequence of *Aspergillus* genera (group II), Table S6: Top 10 threading templates used by I-TASSER, Table S7: Top 10 Identified structural analogs in PDB, Table S8: Best model predicted by I-TASSER, Table S9: Consensus sequence of *Cryptococcus* group, Table S10: Top 10 threading templates used by I-TASSER, Table S11: Top 10 Identified structural analogs in PDB, Table S12: Best model predicted by I-TASSER, Table S13: Human consensus sequence from *Aspergillus* genera (group I), Table S14: Top 10 threading templates used by I-TASSER, Table S15: Top 10 Identified structural analogs in PDB, Table S16: Best model predicted by I-TASSER, Table S17: Human consensus sequence from *Aspergillus* genera (group II), Table S18: Top 10 threading templates used by I-TASSER, Table S19: Top 10 Identified structural analogs in PDB, Table S20: Best model predicted by I-TASSER, Table S21: Human consensus sequence from *Cryptococcus* group, Table S22: Top 10 threading templates used by I-TASSER, Table S23: Top 10 Identified structural analogs in PDB, Table S24: Best model predicted by I-TASSER, Table S25: Conformational analysis of hydrolysis intermediate, Table S26: Scaffold proposals and number of conformations from the conformational analysis calculus, Table S27: Mulliken partial charges for Scaffold A, C and Oxazolinium ion. Scaffold enantiomers showed identical partial charges, Table S28: Rigid docking results in AfChiB1 with the designed ligands, Table S29: Strength of sidechains selected for flexible docking, Table S30: Amino acid residues contribution to **1** in AfChiB1, Table S31: Amino acid residues contribution to **2** in AfChiB1, Table S32: Amino acid residues contribution to **3** in AfChiB1, Table S33: Amino acid residues contribution to **4** in AfChiB1, Table S34: Amino acid residues contribution to **5** in AfChiB1, Table S35: Amino acid residues contribution to **1** in CHIT1, Table S36: Amino acid residues contribution to **2** in CHIT1, Table S37: Amino acid residues contribution to **3** in CHIT1, Table S38: Amino acid residues contribution to **4** in CHIT1, Table S39: Data used for model construction, Table S40: Amino acid residues contribution to Caffeine in AfChiB1, Table S41: Amino acid residues contribution to Pentoxifylline (PTX) in AfChiB1, Table S42: Amino acid residues contribution to Dimethylguanylurea (DGU) in AfChiB1, Table S43: Amino acid residues contribution to Theophylline (TPH) in AfChiB1, Table S44: Amino acid residues contribution to **6** in AfChiB1, Table S45: Amino acid residues contribution to **7** in AfChiB1, Table S46: Amino acid residues contribution to **8** in AfChiB1, Table S47: Energy interaction values (kcal/mol) of the final candidates from flexible docking with AChiBi1 and CHIT1. Candidates aLogP values are also displayed, Table S48: Statistical parameters from model construction, Table S49: Estimation of predicted IC_50_ from equation 1 for ligand **1**–**5**, Table S50: Swiss ADME values for each ligand **1**–**5**.
