# Supplementary material for: Discovery of Octahydroisoindolone as a Scaffold for the Selective Inhibition of Chitinase B1 from Aspergillus fumigatus: In Silico Drug Design Studies
Source: Molecules. 2021 Dec 15;26(24):7606. doi: 10.3390/molecules26247606 (PMC8705689; doi:10.3390/molecules26247606)
Supplement: Supplementary file 1 [file molecules-26-07606-s001.zip › molecules-1498806-supplementary.pdf]

Discovery of octahydroisoindolone as a scaffold for the selective inhibition of chitinase B1 from *Aspergillus fumigatus*: *In-silico* drug design studies

Alberto Marbán-González, [a] Armando Hernández-Mendoza, [b] Mario Ordóñez, [a] Rodrigo Said Razo-Hernández, *[b] José Luis Viveros-Ceballos *[a]

[a] *Centro de Investigaciones Químicas–IICBA, Universidad Autónoma del Estado de Morelos. Av. Universidad 1001, 62209 Cuernavaca, Morelos, México.*
[b] *Centro de Investigación en Dinámica Celular–IICBA, Universidad Autónoma del Estado de Morelos. Av. Universidad 1001, 62209 Cuernavaca, Morelos, México.*

Figure S1. Phylogenetic profile for fungi. 10
Figure S2. Pose of each compound obtained from the re-docking analysis: **1** (green), **2** (yellow), **3** (orange), **4** (brown) and **5** (pink) for AChiB1. 27
Figure S3. Pose of each compound obtained from the re-docking analysis: **1** (green), **2** (yellow), **3** (orange) and **4** (brown) for CHIT1. 28
Figure S4. Structures used for model construction: Caffeine (PDB: 2A3B), Pentoxifylline (PTX; PDB: 2A3C), DGU (PDB: 3CH9), inhibitors **6** (Schüttelkopf et al., 2006), **7** (Jiang et al., 2016), **8** (Jiang et al., 2016), and Theophylline (TPH; PDB: 2A3A) 39

Table S1. Consensus sequence of Aspergillus genera (group I). 3
Table S2. Top 10 threading templates used by I-TASSER. 3
Table S3. Top 10 Identified structural analogs in PDB. 3
Table S4. Best model predicted by I-TASSER. 4
Table S5. Consensus sequence of Aspergillus genera (group II). 4
Table S6. Top 10 threading templates used by I-TASSER. 4
Table S7. Top 10 Identified structural analogs in PDB. 4
Table S8. Best model predicted by I-TASSER. 5
Table S9. Consensus sequence of Cryptococcus group. 5
Table S10. Top 10 threading templates used by I-TASSER. 5
Table S11. Top 10 Identified structural analogs in PDB. 5
Table S12. Best model predicted by I-TASSER. 6
Table S13. Human consensus sequence from Aspergillus genera (group I). 6
Table S14. Top 10 threading templates used by I-TASSER. 6
Table S15. Top 10 Identified structural analogs in PDB. 7
Table S16. Best model predicted by I-TASSER. 7
Table S17. Human consensus sequence from Aspergillus genera (group II). 7
Table S18. Top 10 threading templates used by I-TASSER. 7
Table S19. Top 10 Identified structural analogs in PDB. 8
Table S20. Best model predicted by I-TASSER. 8
Table S21. Human consensus sequence from Cryptococcus group. 8
Table S22. Top 10 threading templates used by I-TASSER. 9

Table S23. Top 10 Identified structural analogs in PDB. 9
Table S24. Best model predicted by I-TASSER. 9
Table S25. Conformational analysis of hydrolysis intermediate 10
Table S26. Scaffold proposals and number of conformations from the conformational analysis calculus. 13
Table S27. Mulliken partial charges for Scaffold **A**, **C** and Oxazolinium ion. Scaffold enantiomers showed identical partial charges. 14
Table S28. Rigid docking results in AfChiB1 with the designed ligands. 15
Table S29. Strength of sidechains selected for flexible docking. 28
Table S30. Amino acid residues contribution to **1** in AfChiB1. 29
Table S31. Amino acid residues contribution to **2** in AfChiB1. 29
Table S32. Amino acid residues contribution to **3** in AfChiB1. 30
Table S33. Amino acid residues contribution to **4** in AfChiB1. 30
Table S34. Amino acid residues contribution to **5** in AfChiB1. 31
Table S35. Amino acid residues contribution to **1** in CHIT1. 31
Table S36. Amino acid residues contribution to **2** in CHIT1. 32
Table S37. Amino acid residues contribution to **3** in CHIT1. 33
Table S38. Amino acid residues contribution to **4** in CHIT1. 33
Table S39. Data used for model construction. 34
Table S40. Amino acid residues contribution to **Caffeine** in AfChiB1. 34
Table S41. Amino acid residues contribution to **Pentoxifylline** (**PTX)** in AfChiB1. 34
Table S42. Amino acid residues contribution to **Dimethylguanylurea** (**DGU)** in AfChiB1. 35
Table S43. Amino acid residues contribution to **Theophylline** (**TPH)** in AfChiB1. 35
Table S44. Amino acid residues contribution to **6** in AfChiB1. 36
Table S45. Amino acid residues contribution to **7** in AfChiB1. 36
Table S46. Amino acid residues contribution to **8** in AfChiB1. 37
Table S47. Energy interaction values (kcal/mol) of the final candidates from flexible docking with AChiBi1 and CHIT1. Candidates aLogP values are also displayed. 38
Table S48. Statistical parameters from model construction. 39
Table S49. Estimation of predicted IC_{50} from equation 1 for ligand **1-5**. 40
Table S50. Swiss ADME values for each ligand **1-5** 40

I-TASSER information about construction of consensus sequence *Aspergillus* genera (group I) 3D structure

Table S1. Consensus sequence of *Aspergillus* genera (group I).

SGYRSVAYFVNWAIYGRNHNPQDLPIDQLTHVLYAFANVRPETGEVYMTDSWADIEKH<br>YPGDSWSDPGNNVYGCIKQMYLLKKKNRNLKVLLSIGGWTYSPNFAPAASTDAGRKNF<br>ADTSVKLLQDLGFDGLDIDWEYPENDQQANDFVLLLKEIRTALDSYSAANAGGQHFLLT<br>VASPAGPDKIKKLHLKDMDAQLDFWNLMAYDYAGSLFSSLTGHQANVYNDTSNPLSTP<br>FNTQTAIDLYLAGGVPANKIVLGMPLYGRSFANTDGLGKPYNGVGQGSWENGVWDYK<br>ALPQAGAEEHVMENIMASYSYDATNKRLISYDNPKVAQLKAGYIKKLGLGGAMWWES<br>SSDKTGTSDSLITTVVNAL

Table S2. Top 10 threading templates used by I-TASSER.

Rank	PDB hit	Iden1	Ident2	Cov	Norm Z-score
1	2a3eA	0.93	0.92	0.99	3.33
2	1d2kA	0.68	0.68	0.99	5.09
3	3chcA	0.93	0.92	0.99	4
4	3g6l	0.55	0.56	0.99	1.75
5	3oa5	0.26	0.29	0.96	1.29
6	3g6lA	0.56	0.56	0.99	3.89
7	3oa5	0.24	0.29	0.98	1.93
8	1w9pA	0.92	0.92	0.99	7.5
9	3g6lA	0.56	0.56	0.99	3.41
10	1w9pA	0.93	0.92	0.99	5.23

Table S3. Top 10 Identified structural analogs in PDB.

Rank	PDB Hit	TM-score	RMSD	IDEN	COV
1	3chcA	0.989	0.51	0.926	0.995
2	1ll6A	0.983	0.72	0.678	0.992
3	3g6lA	0.982	0.7	0.562	0.992
4	1itxA	0.945	1.37	0.373	0.975
5	1ctn_	0.935	1.56	0.326	0.973
6	3b9dA	0.934	1.55	0.321	0.97
7	5dezA2	0.933	1.64	0.329	0.973
8	1rd6A2	0.932	1.65	0.326	0.973
9	1kfwA	0.932	1.6	0.349	0.97
10	3oa5A	0.906	2.02	0.256	0.962

Table S4. Best model predicted by I-TASSER.

Model 1
C-score=1.50
Estimated TM-score = 0.92±0.06
Estimated RMSD = 3.6±2.5Å

I-TASSER information about construction of consensus sequence *Aspergillus* genera (group II) 3D structure

Table S5. Consensus sequence of *Aspergillus* genera (group II).

LQPNPAPTTDTNATIPAMSSGLKSVAYFVNWAIYGRNYNPQDIPADKLTHVLYAFANVR<br>PDSGEVYLSDTWSDIEKHYPTDSWNDVGTNVYGCVKQLFLLKQQNRKLKVLLSIGGWT<br>YSPNFAQAASTDAGRTKFAETATKLVTDLGFDGIDIDWEYPKDDTEAQNMVLLLQKCR<br>ETLDAAAGANRKFLLTIACPAGPANYTKLKLSQMTPYLDFYNLMAYDYAGSWDTVAG<br>HQANLYPSADKPASTPFSTNEAVNYYIQKGGVPSSKIILGMPLYGRAFTNTDGPGTAFSG<br>VGEGSWEQGVWDYKALPRPGATEYVDANLGASWSYDPTARTMVSYDTVAMGEMKLD<br>FITKHQLGGGMWWETSGDKGGKTANKADGSLIGTFVEGIGGV

Table S6. Top 10 threading templates used by I-TASSER.

Rank	PDB hit	Iden1	Ident2	Cov	Norm Z-score
1	2a3eA	0.62	0.59	0.94	3.07
2	1d2kA	0.63	0.6	0.93	5.18
3	3chcA	0.62	0.59	0.94	3.82
4	3g6l	0.58	0.55	0.93	1.71
5	4txg	0.29	0.37	0.99	1.31
6	3g6lA	0.59	0.55	0.93	3.62
7	4dws	0.27	0.33	0.93	1.93
8	1itxA	0.36	0.37	0.94	6.47
9	3g6lA	0.59	0.55	0.93	3.17
10	1w9vA	0.63	0.59	0.94	4.94

Table S7. Top 10 Identified structural analogs in PDB.

Rank	PDB Hit	TM-score	RMSD	IDEN	COV
1	3chcA	0.933	0.66	0.62	0.939
2	1ll7B	0.925	0.56	0.634	0.931
3	3g6mA	0.923	0.65	0.584	0.931
4	1ctn_	0.916	1.71	0.307	0.959
5	3arpA	0.914	1.9	0.293	0.962

6	1rd6A2	0.914	1.69	0.306	0.956
7	5dezA2	0.904	1.78	0.295	0.949
8	1itxA	0.89	1.65	0.36	0.926
9	4txgA	0.875	2.5	0.27	0.941
10	4dwsA	0.87	2.45	0.267	0.939

Table S8. Best model predicted by I-TASSER.

Model 1
C-score=0.98
Estimated TM-score = 0.85±0.08
Estimated RMSD = 4.7±3.1Å

I-TASSER information about construction of consensus sequence *Cryptococcus* group 3D structure.

Table S9. Consensus sequence of *Cryptococcus* group.

FLAVLLLAAIVFLFAQTDFAFPSRPWKAVGEPLKGDEIEMNNPKRTVGYFVNWGIYGRK<br>FFPQNIPGQHLTHINYAFGNVKADSGEVVLSDTWADVEIHYDGDSWDEPPGTNLYGCFK<br>AIYLMKKQNRNLKVLLSIGGWSFSPNFAGIVHPAKWRSTFVQSAVKLVEDVGLDGLDID<br>YEYPKTPRDAEAYVDLLRELRQGLEQLAQSKGKPQGQYQLTVAAPCGWEQMQVLRVR<br>EMDQVLDFWNLMAYDFAGPSWDSVAGHQANLYSDKPDGQATDFSVDRSVRFYLEAG<br>GVHPTKLVIGLPVYGRAFANTKGIGSPFSGTGESAGGSWEAGMWDYKALPQPGTNAQE<br>TNDHRLGASYSYDPAKRLLITYDTQAIAHQKASYIAYHGLGGAMWWELDSDKPEEQTG<br>QSLVRTVREALGGCAQLEWRENELDYPGSKYDNLRRRMEDE

Table S10. Top 10 threading templates used by I-TASSER.

Rank	PDB hit	Iden1	Ident2	Cov	Norm Z-score
1	2a3eA	0.51	0.47	0.88	3.26
2	1d2kA	0.52	0.47	0.87	5.24
3	3chcA	0.52	0.47	0.88	3.9
4	1ll6	0.53	0.47	0.87	1.86
5	3g6l	0.43	0.4	0.87	1.28
6	2a3eA	0.52	0.47	0.88	3.93
7	2a3e	0.52	0.47	0.88	1.97
8	1w9pA	0.51	0.47	0.88	7.82
9	2a3eA	0.51	0.47	0.88	3.33
10	1w9vA	0.51	0.47	0.88	4.9

Table S11. Top 10 Identified structural analogs in PDB.

Rank	PDB Hit	TM-score	RMSD	IDEN	COV

1	3chcA	0.88	0.49	0.514	0.884
2	1ll4D	0.872	0.62	0.52	0.877
3	3g6mA	0.859	0.77	0.443	0.868
4	4txgA	0.79	2.89	0.226	0.859
5	1kfwA	0.787	2.37	0.326	0.837
6	1itxA	0.782	1.67	0.312	0.81
7	1eibA	0.781	2.18	0.283	0.821
8	1rd6A2	0.779	1.99	0.299	0.814
9	3b9eA2	0.778	1.88	0.272	0.812
10	5gztB	0.765	3.27	0.245	0.848

Table S12. Best model predicted by I-TASSER.

Model 1
C-score=0.16
Estimated TM-score = 0.73±0.11
Estimated RMSD = 6.7±4.0Å

I-TASSER information about the construction of human consensus sequence 3D structure from the search in Protein-NCBI webserver for human consensus sequence from *Aspergillus* group I.

Table S13. Human consensus sequence from *Aspergillus* genera (group I).

SAAKLVCYFTNWAQYRQGEARFLPKDLDPSLCTHLIYAFAGMTNHQLSTTEWNDETLY<br>QEFNGLKKMNPKLKTLLAIGGWNFGTQKFTDMVATANNRQTFVNSAIRFLRKYSFDGL<br>DLDWEYPGSQGSPAVDKERFTTLVQDLANAFQQEAQTSGKERLLLSAAVPAGQTYVDA<br>GYEVDKIAQNLDFVNLMAYDFHGSWEKVTGHNSPLYKRQEESGAAASLNVDAAVQQ<br>WLQKGTPASKLILGMPTYGRSFTLASSSDTRVGAPATGSGTPGPFTKEGGMLAYYEVCS<br>WKGATKQRIQDQKVPYIFRDNQWVGFDDVESFKTKVSYLKQKGLGGAMVWALDLDD<br>FAGFSCNQGRYPLIQTLRQEL

Table S14. Top 10 threading templates used by I-TASSER.

Rank	PDB hit	Iden1	Ident2	Cov	Norm Z-score
1	1lq0A	1	0.99	0.99	3.82
2	1vf8A	0.48	0.48	0.99	5.06
3	4wjxA	1	0.99	0.99	4.21
4	1vf8	0.48	0.48	0.99	1.78
5	1vf8	0.48	0.48	0.99	1.3
6	1lq0A	1	0.99	0.99	4.23
7	3fxy	0.58	0.57	0.99	1.99
8	3fy1A	0.58	0.57	0.99	9.15
9	1lq0A	1	0.99	0.99	3.7

10	3fxyA	0.58	0.57	0.99	5.32

Table S15. Top 10 Identified structural analogs in PDB.

Rank	PDB Hit	TM-score	RMSD	IDEN	COV
1	2ybuA	0.994	0.41	0.575	0.997
2	1vf8A	0.991	0.42	0.484	0.995
3	1hkkA	0.991	0.42	0.997	0.995
4	5wupA	0.979	0.72	0.525	0.989
5	5wusA	0.97	0.85	0.475	0.984
6	1la7A	0.97	0.67	0.531	0.978
7	4ay1A	0.958	1.12	0.532	0.981
8	3w4rA	0.957	1.06	0.419	0.978
9	1ljyA	0.956	0.98	0.525	0.973
10	5y2aA	0.947	1.15	0.408	0.97

Table S16. Best model predicted by I-TASSER.

Model 1
C-score=1.82
Estimated TM-score = 0.97±0.05
Estimated RMSD = 3.0±2.1Å

I-TASSER information about the construction of human consensus sequence 3D structure from the search in Protein-NCBI webserver for human consensus sequence from *Aspergillus* group II.

Table S17. Human consensus sequence from *Aspergillus* genera (group II).

VKASQTGFVVLVLLQCCSAYKLVCYYTSWSQYREGDGSCFPDALDRFLCTHIIYSFANIS<br>NDHIDTWEWNDVTLYGMLNTLKNRNPNLKTLLSVGGWNFGSQRFSKIASNTQSRRTFIK<br>SVPPFLRTHGFDGLDLAWLYPGRRDKQHFTTLIKEMKAEFIKEAQPGKKQLLLSAALSA<br>GKVTIDSSYDIAKISQHLDFISIMTYDFHGAWRGTTGHHSPLFRGQEDASPDRFSNTDYA<br>VGYMLRLGAPASKLVMGIPTFGRSFTLASSETGVGAPISGPGIPGRFTKEAGTLAYYEICD<br>FLRGATVHRILGQQVPYATKGNQWVGYDDQESVKSKVQYLKDRQLAGAMVWALDLD<br>DFQGSFCGQDLRFPLTNAIKDAL

Table S18. Top 10 threading templates used by I-TASSER.

Rank	PDB hit	Iden1	Ident2	Cov	Norm Z-score
1	1ljyA	0.82	0.78	0.95	3.57
2	1owqA	0.83	0.79	0.95	5.36

3	4ay1A	0.52	0.49	0.95	3.84
4	1vf8	0.47	0.45	0.95	1.76
5	3fxy	0.51	0.49	0.95	1.28
6	1ljyA	0.82	0.78	0.95	4.03
7	4ay1	0.52	0.49	0.94	1.92
8	3fy1A	0.52	0.49	0.95	8.06
9	4ay1A	0.52	0.49	0.95	3.58
10	4ay1A	0.52	0.49	0.95	5.13

Table S19. Top 10 Identified structural analogs in PDB.

Rank	PDB Hit	TM-score	RMSD	IDEN	COV
1	5wupA	0.946	0.71	0.446	0.955
2	2ybuA	0.943	0.61	0.515	0.95
3	1owqA	0.941	0.57	0.835	0.947
4	1vf8A	0.938	0.69	0.469	0.947
5	1nwtA	0.939	0.92	1	0.95
6	1hkkA	0.938	0.61	0.549	0.944
7	5wusA	0.934	0.84	0.441	0.947
8	4ay1A	0.934	0.95	0.518	0.95
9	3w4rA	0.915	1.05	0.365	0.934
10	5y29A	0.906	1.45	0.448	0.934

Table S20. Best model predicted by I-TASSER.

Model 1
C-score=0.22
Estimated TM-score = 0.74±0.11
Estimated RMSD = 6.2±3.8Å

I-TASSER information about the construction of human consensus sequence 3D structure from the search in Protein-NCBI webserver for the human consensus sequence from *Cryptococcus* group.

Table S21. Human consensus sequence from *Cryptococcus* group.

MTKLILLTGLVLILNLQLGSAYQLTCYFTNWAQYRPGLGRFMPDNIDPCLCTHLIYAFAG<br>RQNNEITTIEWNDVTLYQAFNGLKNKNSQLKTLLAIGGWNFGTAPFTAMVSTPENRQTF<br>ITSVIKFLRQYEFDGLDFDWEYPGSRGSPPQDKHLFTVLVQEMREAFEQEAKQINKPRLM<br>VTAAVAAGISNIQSGYEIPQLSQYLDYIHVMTYDLHGSWEGYTGENSPLYKYPTDTGSN<br>AYLNVDYVMNYWKDNGAPAEKLIVGFPTYGHNFILSNPSNTGIGAPTSGAGPAGPYAKE<br>SGIWAYYEICTFLKNGATQGWDAPQEVPYAYQGNVWVGYDNVKSFDIKAQWLKHNKF<br>GGAMVWAIDLDDFTGTFCNQGKFPLISTLKKALGLQSASCTAPAQPIEPITAAPSGS

Table S22. Top 10 threading templates used by I-TASSER.

Rank	PDB hit	Iden1	Ident2	Cov	Norm Z-score
1	3fxyA	0.99	0.92	0.92	3.46
2	3fxyA	0.99	0.92	0.92	5.23
3	3fy1A	0.99	0.91	0.92	4.01
4	3fxy	0.99	0.92	0.92	1.88
5	3fxy	0.99	0.92	0.92	1.33
6	3fxyA	0.99	0.92	0.92	4.02
7	3fxy	0.99	0.92	0.92	2.04
8	3fy1A	0.99	0.91	0.92	11.52
9	5wusA	0.47	0.43	0.91	3.53
10	3fxyA	0.99	0.92	0.92	5.22

Table S23. Top 10 Identified structural analogs in PDB.

Rank	PDB Hit	TM-score	RMSD	IDEN	COV
1	3fxyA	0.921	0.29	0.992	0.922
2	1vf8A	0.902	0.6	0.686	0.907
3	5wupA	0.884	0.93	0.486	0.895
4	1hkjA	0.883	0.57	0.573	0.888
5	5wusA	0.875	1.07	0.468	0.888
6	1la7A	0.872	0.75	0.511	0.881
7	3w4rA	0.862	1.25	0.428	0.883
8	4ay1A	0.859	1.24	0.506	0.881
9	1ljyA	0.858	1.21	0.522	0.876
10	5y2aA	0.857	1.3	0.392	0.878

Table S24. Best model predicted by I-TASSER.

Model 1
C-score=0.31
Estimated TM-score = 0.75±0.10
Estimated RMSD = 6.2±3.8Å

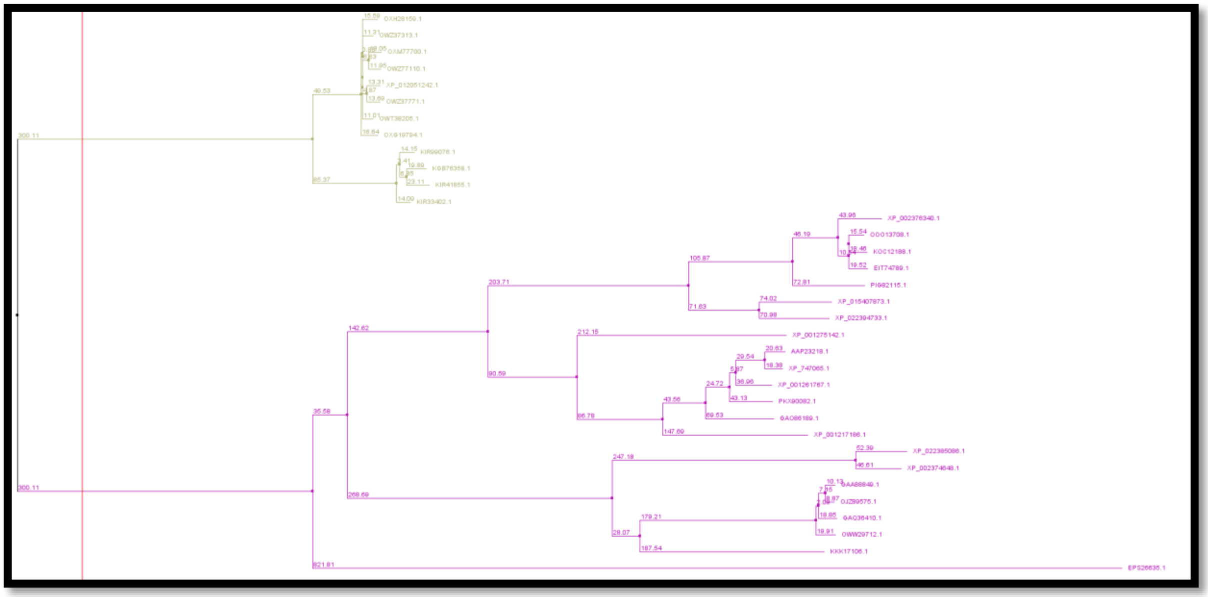

Figure S1. Phylogenetic profile for fungi.

Table S25. Conformational analysis of hydrolysis intermediate

Intermediate conformers	Structure conformation	Energy (kJ/mol)
A	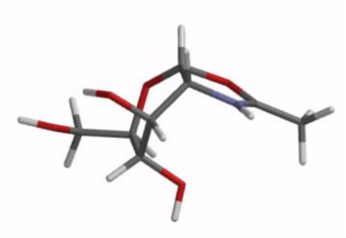	-1952002.64
B	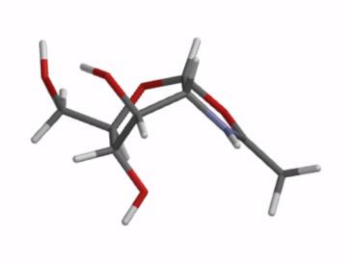	-1952010.23
C	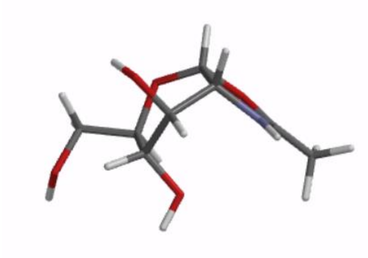	-1951986.01

D	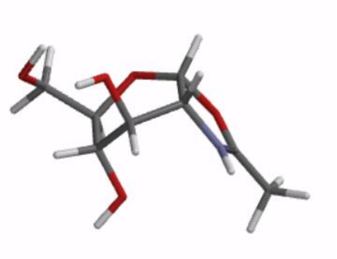	-1951987.78
E	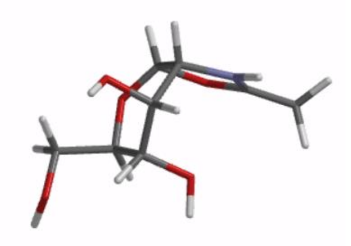	-1951995.24
F	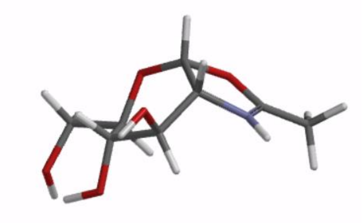	-1951979.21
G	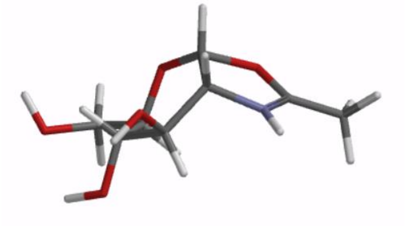	-1951977.23
H	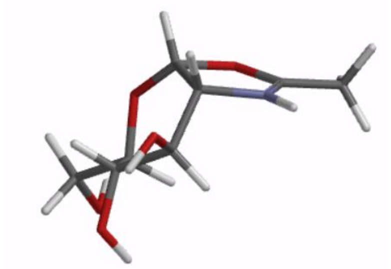	-1951958.48
I	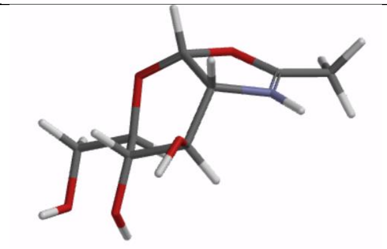	-1951960.02

J	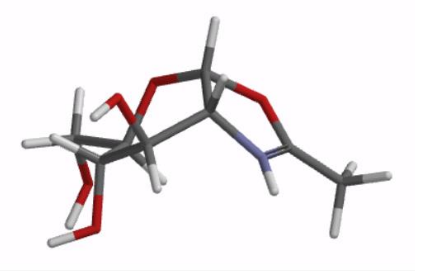	-1951948.75
K	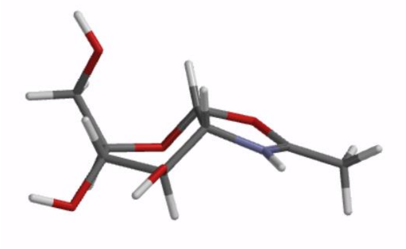	-1951994.91
L	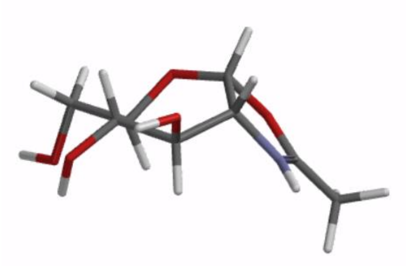	-1951968.33
M	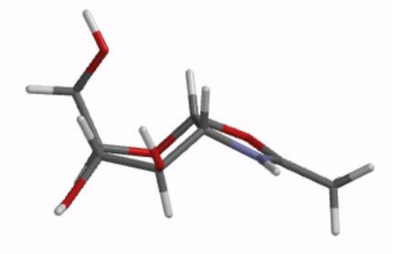	-1951982.77
N	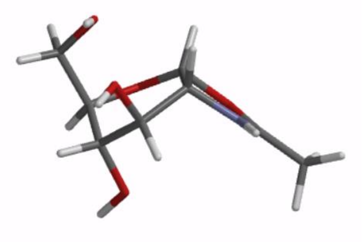	-1951981.67
O	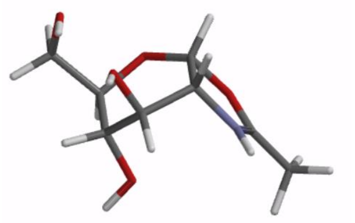	-1951973.33

P	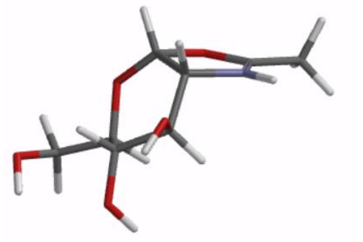	-1951959.59
Q	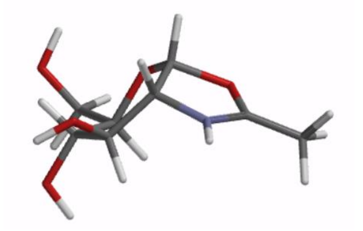	-1951966.95
R	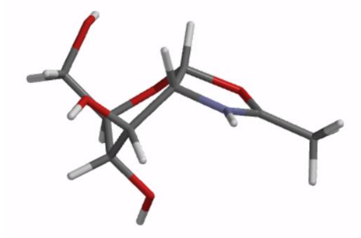	-1951945.88
S	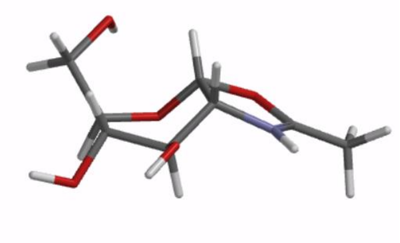	-1951948.95

Table S26. Scaffold proposals and number of conformations from the conformational analysis calculus.

Scaffold name	Structure	Number of conformations
A	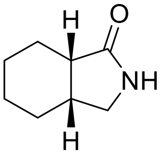	4
ent-A	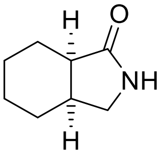	4

B	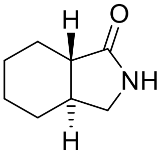	1
ent-B	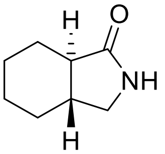	2
C	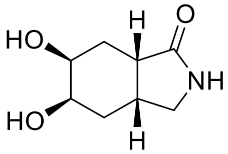	14
ent-C	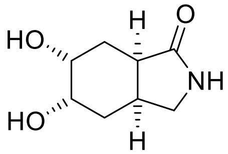	16
D	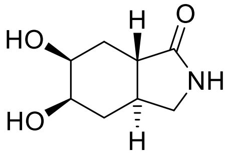	5
ent-D	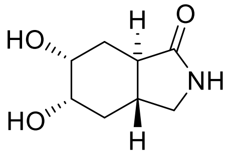	6
E	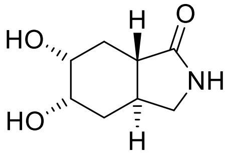	9
ent-E	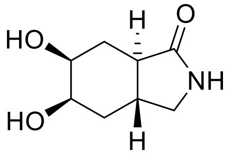	8

Table S27. Mulliken partial charges for Scaffold **A**, **C** and Oxazolinium ion. Scaffold enantiomers showed identical partial charges.

Atom	A	C	Oxazolinium ion
C(sp^2)	0.277	0.23	0.599
O	-0.510	-0.494	-0.245
N	-0.483	-0.477	-0.460

Table S28. Rigid docking results in AfChiB1 with the designed ligands.

Ligand	Number of found Poses in separated runs	Mean MolDock score (kcal/mol)	aLogP
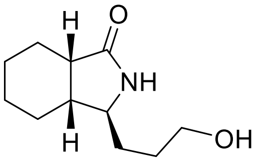	2	-58.15335	1.08
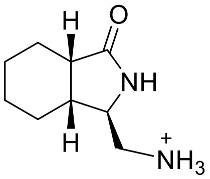	3	-80.36856667	-0.01
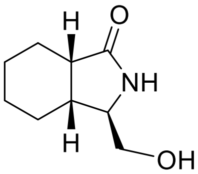	3	-65.4547	0.19
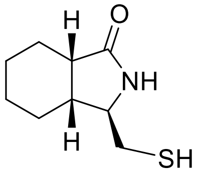	3	-64.87576667	1.47
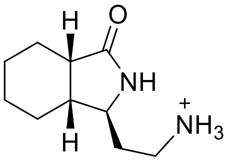	2	-81.25555	0.41
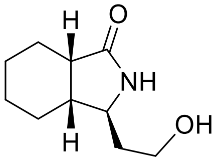	3	-69.3732	0.62
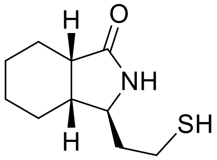	3	-63.89726667	1.83
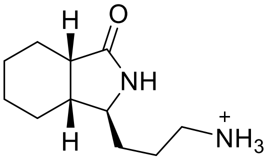	3	-73.7296	0.77
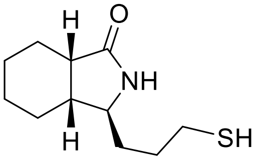	2	-52.89545	2.27

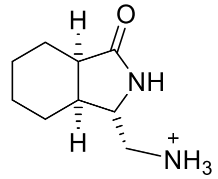	4	-77.735575	-0.01
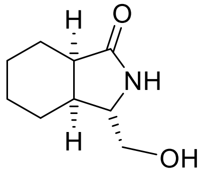	0	-68.5139	0.19
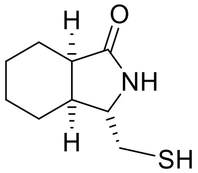	3	-68.63076667	1.47
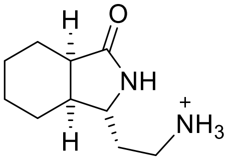	1	-70.7717	0.41
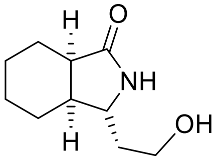	2	-61.2393	0.62
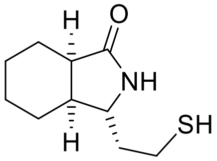	4	-65.3343	1.83
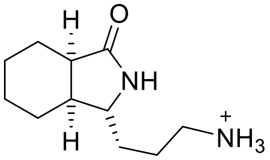	2	-67.69365	0.77
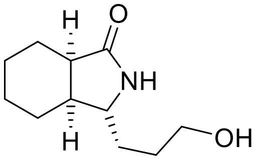	2	-49.81995	1.08
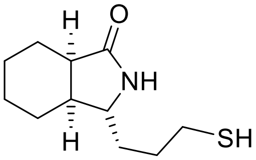	2	-50.85655	2.27
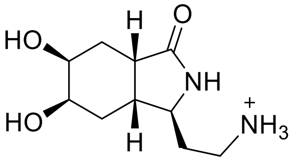	3	-88.82263333	-1.56

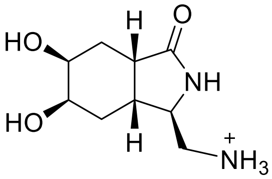	4	-88.180475	-2
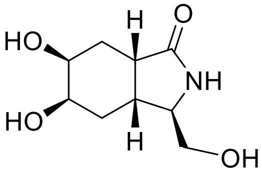	4	-74.706325	-1.8
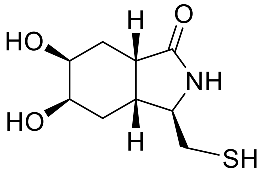	3	-71.47643333	-0.21
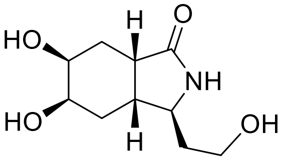	4	-71.7987	-1.56
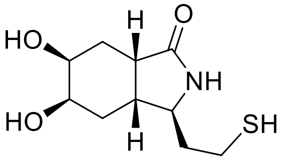	4	-79.5695	0.11
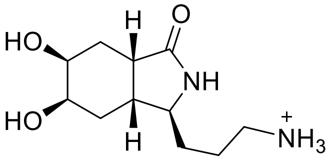	0	Not found	-1.18
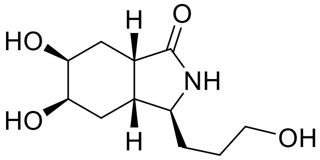	0	Not found	-1.17
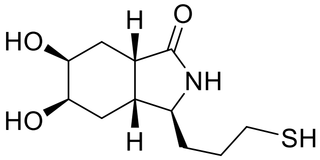	1	-81.9783	0.46
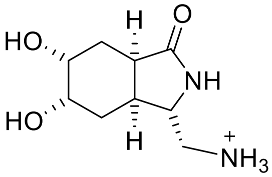	4	-78.4431	-2

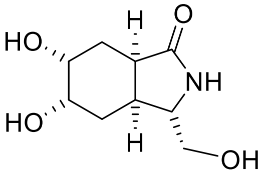	4	-68.871575	-1.8
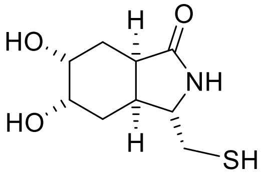	4	-73.36815	-0.21
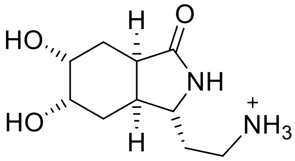	4	-82.297575	-1.56
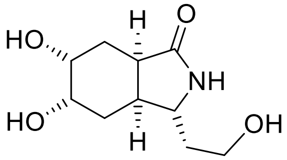	4	-77.058925	-1.56
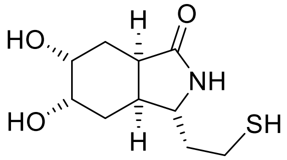	4	-79.91665	0.11
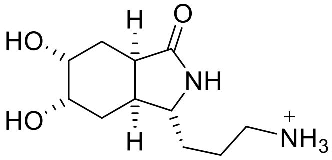	4	-93.896625	-1.18
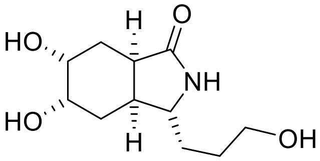	4	-91.234775	-1.17
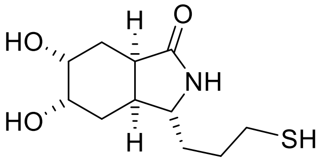	4	-86.68	0.46
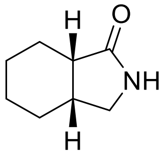	4	-52.0628	0.82
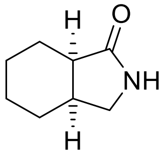	4	-58.341275	0.82

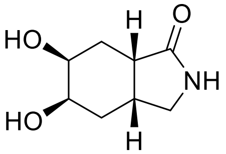	4	-56.3282	-1.32
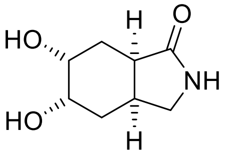	4	-68.1698	-1.32
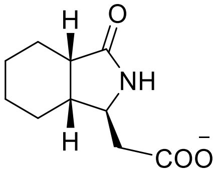	4	-64.449275	0.8
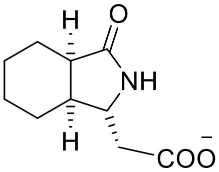	1	-69.8683	0.8
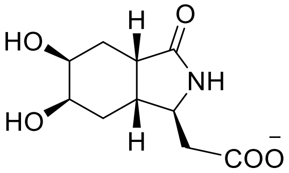	1	-53.025	-1.67
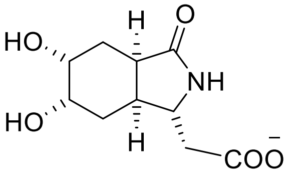	4	-78.412325	-1.67
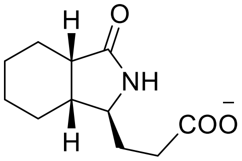	3	-56.86323333	1.09
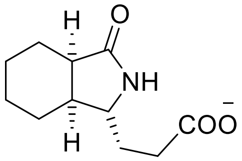	3	-54.12516667	1.09
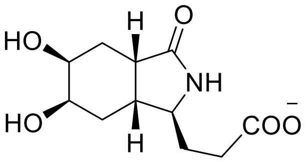	0	Not found	-1.41
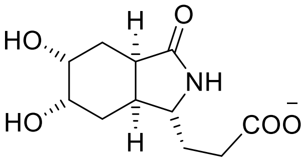	4	-87.9446	-1.41

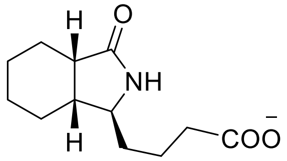	3	-60.63966667	1.49
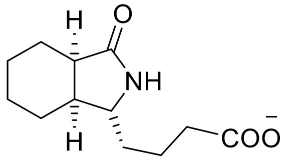	3	-50.7148	1.49
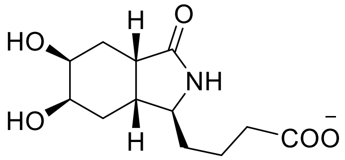	0	Not found	-1.06
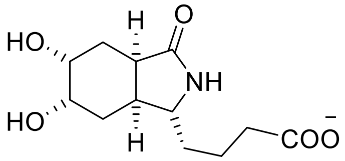	4	-91.478575	-1.06
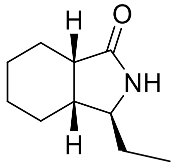	4	-65.814475	2.1
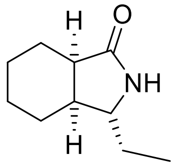	4	-74.801675	2.1
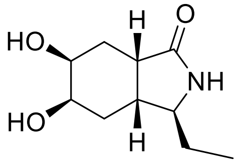	3	-68.8856	-0.58
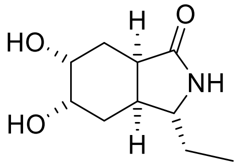	4	-72.21065	-0.58
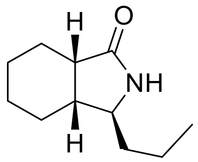	2	-70.23215	2.57
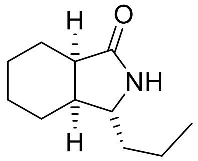	4	-74.173575	2.57

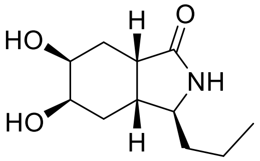	3	-70.90633333	-0.23
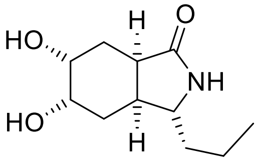	4	-81.051075	-0.23
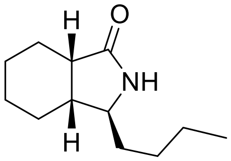	3	-63.67743333	3.08
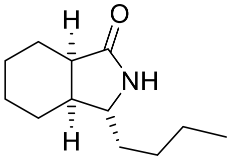	4	-81.32605	3.08
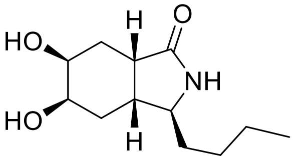	2	-78.84105	0.27
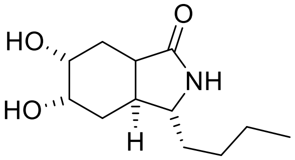	4	-86.0773	0.27
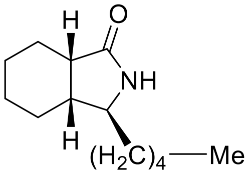	3	-48.80823333	3.58
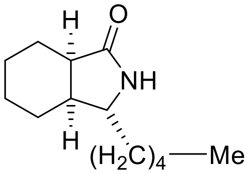	4	-84.208575	3.58
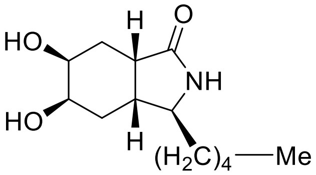	0	Not found	0.64

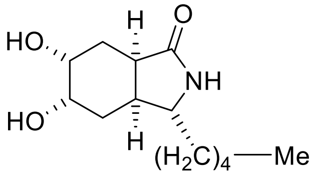	4	-90.64115	0.64
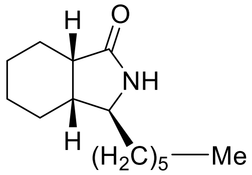	0	Not found	4.14
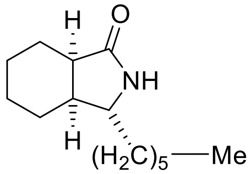	1	-96.3333	4.14
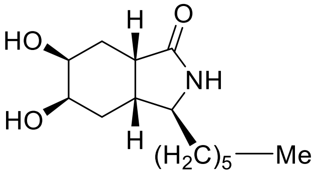	1	Not found	1.12
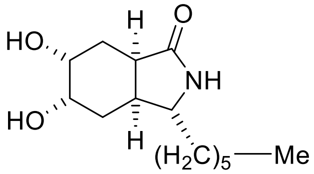	3	-107.89175	1.12
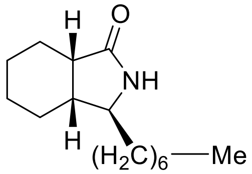	0	Not found	4.69
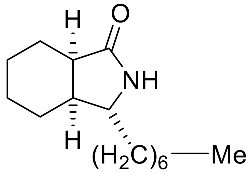	0	Not found	4.69
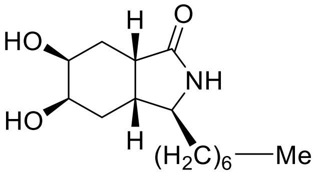	0	Not found	1.6
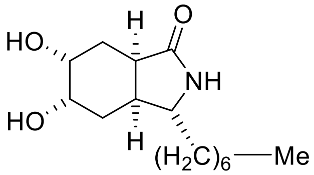	3	-107.5303333	1.6

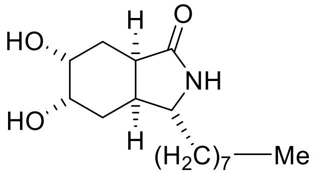	2	-94.49845	2.23
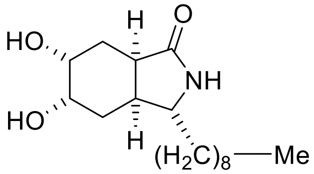	2	-95.1704	2.78
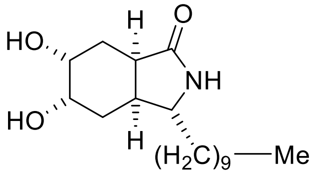	4	-106.76705	3.31
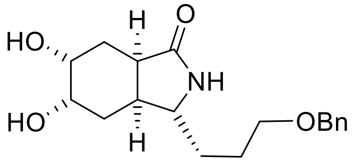	1	-90.5388	0.65
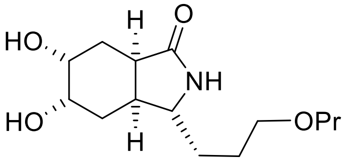	2	-109.885	0.12
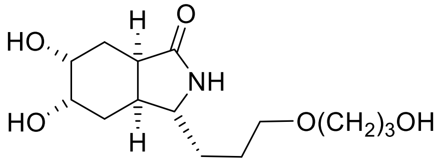	1	-115.376	-1.12
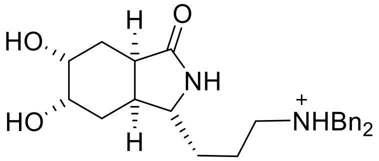	3	-122.93	2.28
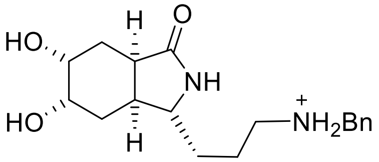	2	-122.5165	1.11
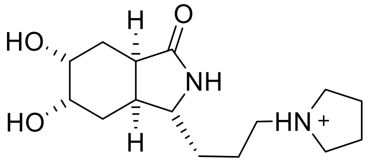	3	-112.9103333	0.44
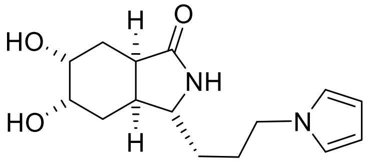	4	-114.8825	0.29

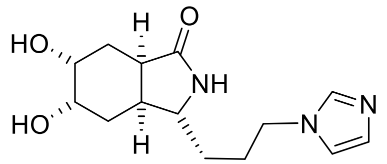	0	Not found	-0.52
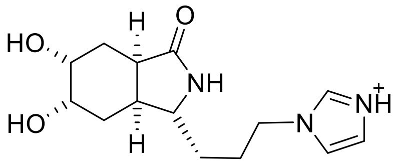	0	Not found	-0.52
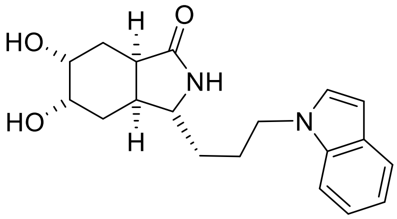	0	Not found	1.65
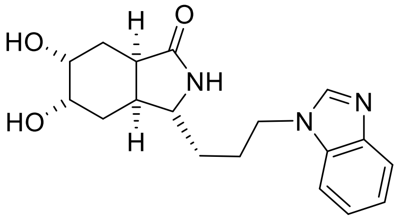	0	Not found	0.72
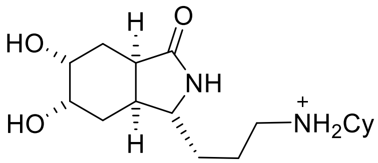	3	-101.5601333	1.14
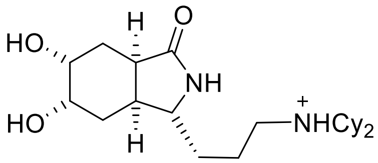	1	-107.622	3.69
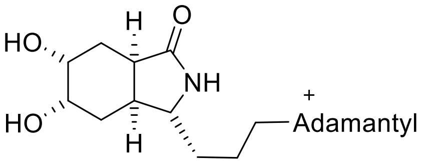	1	-103.583	2.04
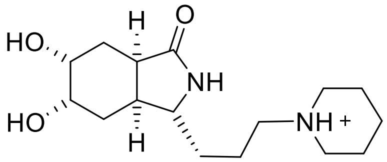	0	Not found	0.81
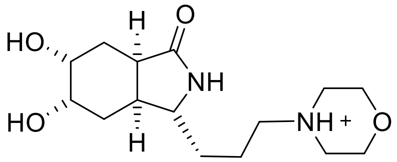	3	-120.147	-0.36

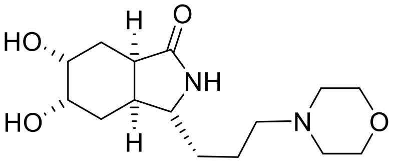	0	Not found	-0.36
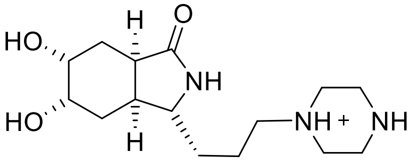	4	-120.207	-1.17
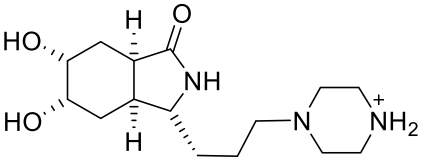	0	Not found	-1.17
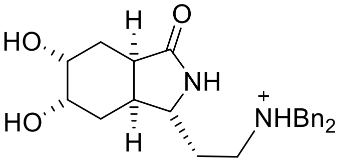	0	Not found	1.92
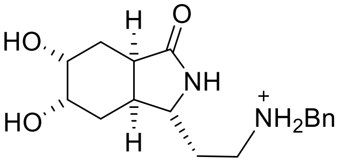	4	-123.31375	0.25
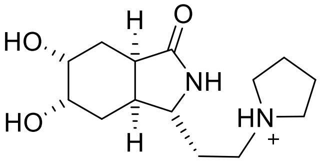	4	-102.02625	-0.12
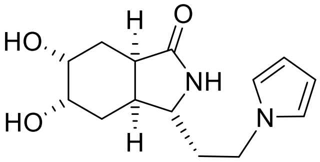	3	-103.627	0.05
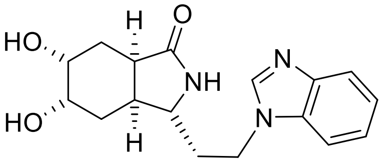	0	Not found	0.38
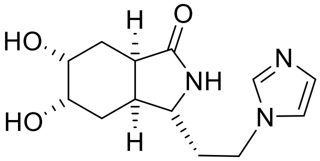	0	Not found	-0.81
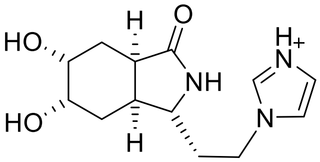	0	Not found	-0.81

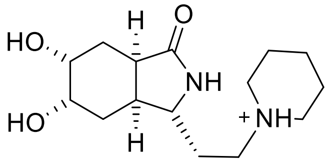	0	Not found	0.51
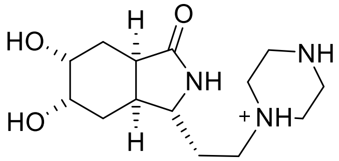	4	-107.4735	-1.51
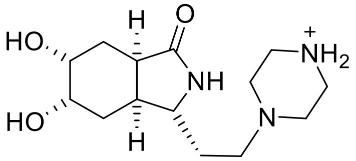	0	Not found	-1.51
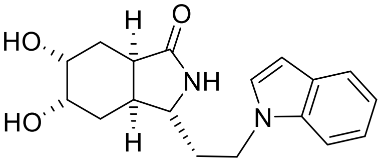	0	Not found	1.33
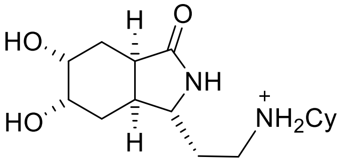	4	-123.23925	0.66
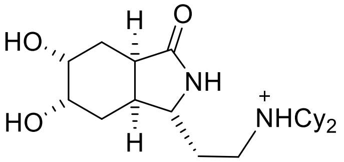	0	Not found	4.05
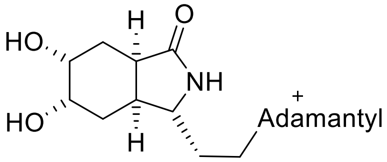	0	Not found	1.68
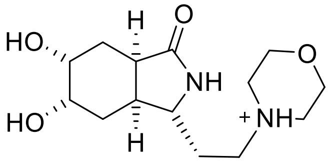	0	Not found	-0.79
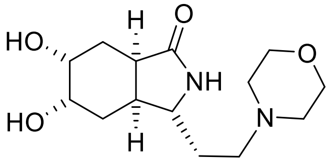	0	Not found	-0.79
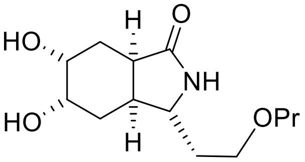	4	-96.82515	-0.09

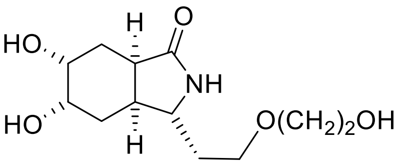	0	Not found	-1.46
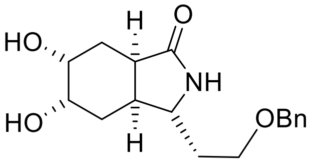	2	-123.233	0.39
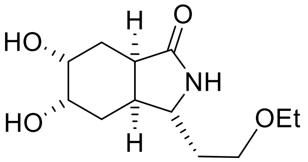	4	-91.450875	-0.63
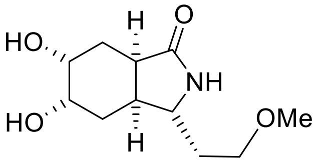	4	-87.2996	-1.01
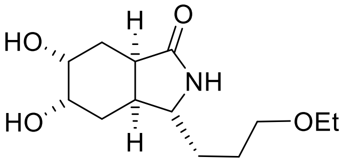	4	-109.0805	-0.14
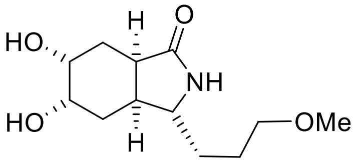	4	-98.42235	-0.72

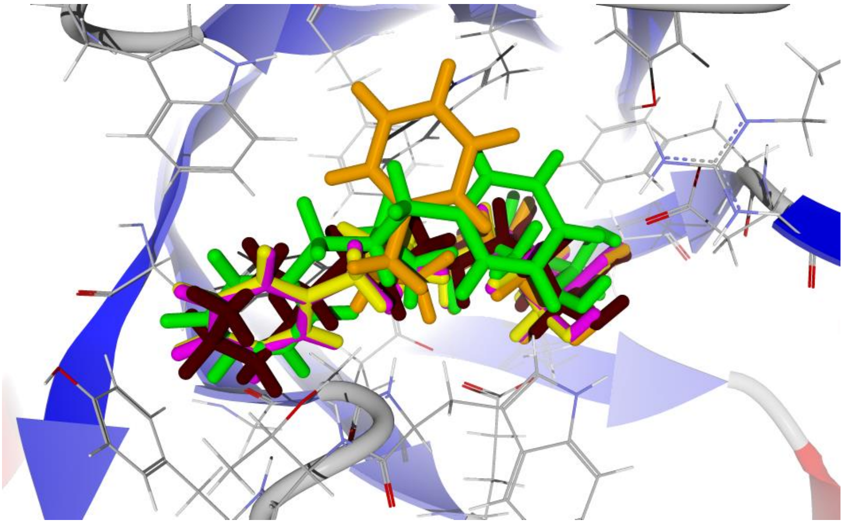

Figure S2. Pose of each compound obtained from the re-docking analysis: **1** (green), **2** (yellow), **3** (orange), **4** (brown) and **5** (pink) for AChiB1.

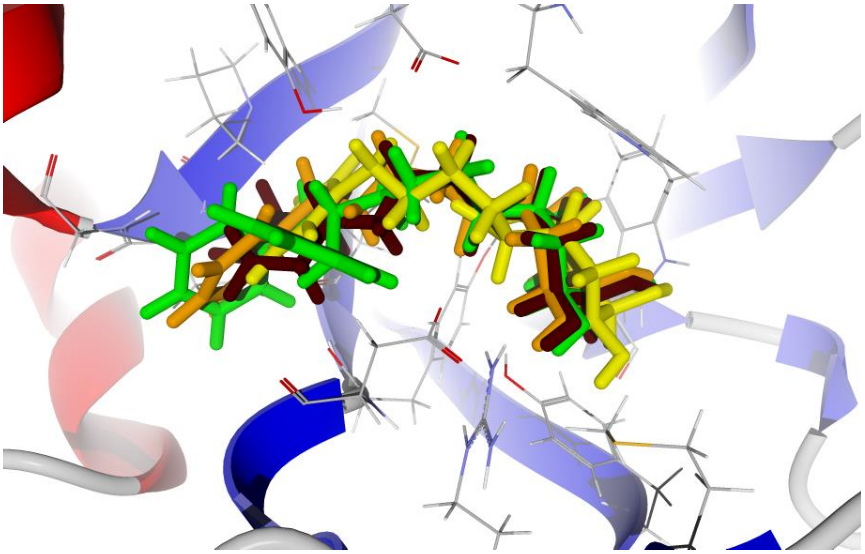

Figure S3. Pose of each compound obtained from the re-docking analysis: **1** (green), **2** (yellow), **3** (orange) and **4** (brown) for CHIT1.

Table S29. Strength of sidechains selected for flexible docking.

Residues in AfChiB1	Strength	Residues in CHIT1	Strength
Trp52	1	Trp31	0
Arg57	0	Phe58	0
Phe76	1	Trp99	0
Trp137	1	Asp138	0.5
Asp175	0.5	Gu140	0.5
Glu177	0.5	Tyr141	1
Tyr178	1	Tyr190	1
Lys224	0	Met210	1
Met 243	1	Asp213	0.5
Arg301	0	Arg269	0
Trp384	1	Asp213	0

Table S30. Amino acid residues contribution to **1** in AfChiB1.

Residue	ID	Total	EPair	EElec (r > 4.5)	EElec (r < 4.5)
Trp	384	-29.0818	-29.0818		
Trp	137	-17.527	-17.527		
Phe	76	-15.7162	-15.7162		
Glu	177	-13.4926	-10.4461	-1.03823	-2.0083
Thr	138	-12.1323	-12.1323		
Trp	52	-11.5397	-11.5397		
Tyr	139	-10.8028	-10.8028		
Asp	246	-10.0862	-9.50607	-1.5513	0.971138
Asp	175	-7.56112	-7.4445	-1.1953	1.07868
Gly	136	-7.5581	-7.5581		
Met	243	-6.69166	-6.69166		
Tyr	245	-5.18425	-5.18425		
Tyr	48	-5.15289	-5.15289		
Glu	322	-5.13877	-3.32816	-1.8106	
Gly	135	-2.16696	-2.16696		
Asp	385	-0.98667	0	-0.98667	
Ala	217	-0.966115	-0.966115		
Tyr	299	-0.805855	-0.805855		
Tyr	178	-0.583219	-0.583219		
Met	382	-0.445702	-0.445702		
Arg	57	0.688037	0	0.688037	

Table S31. Amino acid residues contribution to **2** in AfChiB1.

Residue	ID	Total	EPair	EElec (r > 4.5)	EElec (r < 4.5)
Trp	384	-21.1793	-21.1793		
Trp	137	-16.9929	-16.9929		
Phe	76	-16.4578	-16.4578		
Thr	138	-12.5796	-12.5796		
Tyr	139	-10.6595	-10.6595		
Glu	177	-9.84444	-8.02492	-1.06017	-0.759355
Tyr	245	-9.51937	-9.51937		
Trp	52	-9.02308	-9.02308		
Asp	175	-8.15766	-7.43463	-0.605857	-0.117174
Gly	136	-8.05947	-8.05947		
Asp	246	-5.27243	-4.97304	-0.805846	0.506462
Tyr	48	-5.08551	-5.08551		
Met	243	-4.63916	-4.63916		
Gly	135	-1.55817	-1.55817		

Glu	322	-1.35444	0	-1.35444	
Ala	217	-0.905758	-0.905758		
Asp	385	-0.876229	0	-0.876229	
Tyr	178	-0.830515	-0.830515		
Met	382	-0.510592	-0.510592		
Arg	57	0.6738	0	0.6738	
Arg	301	0.851369	0	0.851369	

Table S32. Amino acid residues contribution to **3** in AfChiB1.

Residue	ID	Total	EPair	EElec (r > 4.5)	EElec (r < 4.5)
Trp	384	-30.4486	-30.4486		
Trp	137	-14.2574	-14.2574		
Glu	177	-11.6066	-7.32135	-2.27611	-2.00913
Tyr	245	-9.5998	-9.5998		
Asp	175	-7.64883	-6.82407	-0.147902	-0.676867
Phe	76	-6.20945	-6.20945		
Glu	322	-5.79197	-3.9232	-1.52883	-0.339936
Met	243	-5.30891	-5.30891		
Trp	52	-5.26909	-5.26909		
Asp	246	-5.03691	-4.49123	-1.57314	1.02746
Tyr	48	-4.86504	-4.86504		
Gly	136	-3.86255	-3.86255		
Asp	385	-1.68281	-0.515937	-1.16688	
Thr	138	-1.31347	-1.31347		
Tyr	178	-0.793236	-0.793236		
Ala	217	-0.78956	-0.78956		
Met	382	-0.496319	-0.496319		
Val	50	-0.443876	-0.443876		
Arg	57	-0.434485	-0.434485		
Arg	301	0.462819	0	0.462819	

Table S33. Amino acid residues contribution to **4** in AfChiB1.

Residue	ID	Total	EPair	EElec (r > 4.5)	EElec (r < 4.5)
Trp	384	-21.9013	-21.9013		
Trp	137	-15.2879	-15.2879		
Phe	76	-12.8413	-12.8413		
Glu	177	-11.786	-8.28824	-0.767571	-2.73016
Thr	138	-11.0739	-11.0739		
Tyr	139	-10.7439	-10.7439		

Asp	246	-9.11196	-8.66937	-1.38043	0.937835
Tyr	245	-8.53967	-8.53967		
Trp	52	-7.57687	-7.57687		
Gly	136	-7.47833	-7.47833		
Met	243	-5.47695	-5.47695		
Asp	175	-4.74932	-4.12859	-2.43385	1.81312
Tyr	48	-3.25918	-3.25918		
Gly	135	-2.10734	-2.10734		
Glu	322	-1.23188	0	-1.23188	
Asp	385	-0.910039	0	-0.910039	
Tyr	178	-0.823499	-0.823499		
Ala	217	-0.528891	-0.528891		
Arg	57	0.637264	0	0.637264	
Arg	301	0.833902	-0.0258415	0.859744	

Table S34. Amino acid residues contribution to **5** in AfChiB1.

Residue	ID	Total	EPair	EElec (r > 4.5)	EElec (r < 4.5)
Trp	384	-21.4893	-21.4893		
Trp	137	-16.645	-16.645		
Phe	76	-15.6587	-15.6587		
Thr	138	-12.798	-12.798		
Tyr	139	-10.9705	-10.9705		
Glu	177	-9.90266	-7.69997	-0.375085	-1.8276
Tyr	245	-9.82267	-9.82267		
Trp	52	-8.83312	-8.83312		
Gly	136	-8.21529	-8.21529		
Asp	175	-7.31786	-7.54054	1.31687	-1.09419
Met	243	-5.23135	-5.23135		
Tyr	48	-5.03628	-5.03628		
Asp	246	-3.58222	-4.36767	0.037445	0.748006
Gly	135	-1.47181	-1.47181		
Ala	217	-0.85295	-0.85295		
Tyr	178	-0.735283	-0.735283		
Met	382	-0.518115	-0.518115		

Table S35. Amino acid residues contribution to **1** in CHIT1.

Residue	ID	Total	EPair	EElec (r > 4.5)	EElec (r < 4.5)
Asp	213	-33.4181	-29.0185	-1.03325	-3.3664
Trp	99	-27.8759	-27.8759		
Trp	358	-17.3255	-17.3255		
Tyr	212	-14.6678	-14.6678		
Met	210	-12.7853	-12.7853		

Tyr	141	-11.3983	-11.3983		
Tyr	267	-11.2183	-11.2183		
Ala	186	-10.1261	-10.1261		
Phe	214	-8.8203	-8.8203		
Trp	218	-8.29445	-8.29445		
Tyr	190	-6.65345	-6.65345		
Glu	140	-5.85524	-3.43638	-2.51199	0.093123
Pro	185	-5.66524	-5.66524		
Gly	187	-4.33288	-4.33288		
Asp	138	-0.755269	-0.364759	-0.39051	
Glu	219	-0.717877	0	-0.717877	
Leu	241	-0.536074	-0.536074		
Met	300	-0.459899	-0.459899		
Leu	362	-0.31709	-0.31709		
Lys	253	0.377347	0	0.377347	
Lys	343	0.446324	0	0.446324	

Table S36. Amino acid residues contribution to **2** in CHIT1.

Residue	ID	Total	EPair	EElec (r > 4.5)	EElec (r < 4.5)
Trp	99	-33.1063	-33.1063		
Asp	213	-24.9518	-20.7377	-3.22875	-0.985356
Trp	358	-15.4112	-15.4112		
Tyr	267	-14.1911	-14.1911		
Glu	140	-13.3553	-7.53858	-1.40943	-4.40728
Met	210	-12.1978	-12.1978		
Tyr	141	-9.54924	-9.54924		
Tyr	212	-9.01046	-9.01046		
Pro	185	-5.66591	-5.66591		
Ala	186	-3.57854	-3.57854		
Val	184	-1.41866	-1.41866		
Asp	138	-0.999762	-0.14069	-0.859072	
Tyr	190	-0.911348	-0.911348		
Gly	187	-0.710759	-0.710759		
Leu	362	-0.63994	-0.63994		
Glu	297	-0.348929	-0.00184695	-0.347082	
Lys	253	0.339007	0	0.339007	
Lys	343	0.432074	0	0.432074	
Arg	269	0.551869	0	0.551869	
Met	300	1.99947	1.99947		

Table S37. Amino acid residues contribution to **3** in CHIT1.

Residue	ID	Total	EPair	EElec (r > 4.5)	EElec (r < 4.5)
Trp	99	-22.4892	-22.4892		
Asp	213	-20.8316	-17.4773	-1.90671	-1.44763
Trp	358	-18.8656	-18.8656		
Tyr	212	-17.3011	-17.3011		
Met	210	-13.5573	-13.5573		
Tyr	267	-10.2605	-10.2605		
Glu	140	-9.71609	-6.19552	-2.20811	-1.31246
Phe	214	-7.16865	-7.16865		
Ala	186	-6.61337	-6.61337		
Tyr	141	-5.63077	-5.63077		
Pro	185	-5.15822	-5.15822		
Gly	187	-3.28651	-3.28651		
Tyr	190	-2.52143	-2.52143		
Asp	138	-1.40696	-0.509549	-0.897406	
Met	300	-1.30312	-1.30312		
Glu	219	-0.616962	0	-0.616962	
Lys	253	0.368472	0	0.368472	
Lys	343	0.488932	0	0.488932	
Arg	269	0.864277	0	0.864277	

Table S38. Amino acid residues contribution to **4** in CHIT1.

Residue	ID	Total	EPair	EElec (r > 4.5)	EElec (r < 4.5)
Asp	213	-28.8588	-22.2599	-0.683194	-5.91573
Trp	99	-25.8435	-25.8435		
Trp	358	-18.6255	-18.6255		
Tyr	212	-17.2824	-17.2824		
Met	210	-14.8065	-14.8065		
Tyr	267	-11.3155	-11.3155		
Glu	140	-8.65371	-4.8129	-1.2326	-2.60821
Pro	185	-4.89694	-4.89694		
Tyr	141	-4.83949	-4.83949		
Ala	186	-3.10926	-3.10926		
Gly	187	-1.53145	-1.53145		
Phe	214	-1.18177	-1.18177		
Asp	138	-1.16051	-0.263148	-0.897366	
Val	184	-1.05398	-1.05398		
Met	300	-0.482245	-0.482245		
Tyr	190	-0.312125	-0.312125		

Glu	219	-0.300206	0	-0.300206	
Lys	253	0.337956	0	0.337956	
Arg	269	0.42259	-0.983544	1.92331	-0.517179
Lys	343	0.476754	0	0.476754	

Table S39. Data used for model construction.

Ligand	IC_{50}	MolDock score	aLogP
**Caffeine**	469	-68.3453	-1.78
**PTX**	126	-129.992	-1
**THP**	1500	-67.5685	-1.22
**Dicaffeine**	4.8	-185.573	-1.26
**DGU**	500	-68.2	-1.05
**6**	520	-125.137	2.07
**7**	92.9	-154.145	3.13
**8**	51.8	-175.6	4.19

Table S40. Amino acid residues contribution to **Caffeine** in AfChiB1.

Residue	ID	Total	EPair
Trp	137	-14.7543	-14.7543
Glu	177	-12.9188	-12.9188
Tyr	245	-11.7931	-11.7931
Trp	384	-10.2368	-10.2368
Asp	246	-7.67069	-7.67069
Met	243	-6.90224	-6.90224
Asp	175	-4.06906	-4.06906
Phe	76	-2.28276	-2.28276
Gly	136	-1.30334	-1.30334
Tyr	178	-1.12867	-1.12867

Table S41. Amino acid residues contribution to **Pentoxifylline** (**PTX)** in AfChiB1.

Residue	ID	Total	EPair	EElec (r > 4.5)	EElec (r < 4.5)
Trp	137	-19.4407	-19.4407		
Trp	384	-12.4779	-12.4779		
Glu	177	-11.847	-11.4025	1.68571	-2.1302
Asp	246	-11.7394	-12.2205	-0.738627	1.21976
Met	243	-9.04744	-9.04744		
Asp	175	-8.44357	-7.60456	-0.574233	-0.264776
Tyr	48	-7.31853	-7.31853		
Arg	301	-5.1076	-3.90797	-0.622204	-0.577419
Phe	251	-3.82638	-3.82638		

Tyr	245	-3.68348	-3.68348		
Gly	136	-2.85439	-2.85439		
Ala	217	-0.774556	-0.774556		
Met	382	-0.54296	-0.54296		
Tyr	178	-0.454263	-0.454263		
Phe	76	0.355588	0.355588		

Table S42. Amino acid residues contribution to **Dimethylguanylurea** (**DGU)** in AfChiB1.

Residue	ID	Total	EPair	EElec (r > 4.5)	EElec (r < 4.5)
Trp	384	-13.0256	-13.0256		
Trp	137	-9.42954	-9.42954		
Glu	177	-8.57023	-9.06635	1.03895	-0.542824
Tyr	245	-6.31521	-6.31521		
Met	243	-5.12098	-5.12098		
Tyr	48	-4.24297	-4.24297		
Asp	175	-3.97196	-4.21733	-0.376202	0.621571
Phe	76	-3.24909	-3.24909		
Asp	246	-1.65218	-1.37531	0.415706	-0.692578
Gly	136	-0.899272	-0.899272		
Ala	217	-0.720333	-0.720333		

Table S43. Amino acid residues contribution to **Theophylline** (**TPH)** in AfChiB1.

Residue	ID	Total	EPair
Trp	137	-13.2897	-13.2897
Glu	177	-12.6916	-12.6916
Trp	384	-12.0463	-12.0463
Met	243	-7.21599	-7.21599
Asp	175	-6.70417	-6.70417
Tyr	245	-4.72696	-4.72696
Asp	246	-3.80021	-3.80021
Tyr	48	-3.72834	-3.72834
Gly	136	-3.36767	-3.36767
Phe	76	-2.97139	-2.97139
Ala	217	-0.500805	-0.500805

Table S44. Amino acid residues contribution to **6** in AfChiB1.

Residue	ID	Total	EPair	EElec (r > 4.5)	EElec (r < 4.5)
Trp	137	-34.9374	-34.9374		
Phe	251	-16.5709	-16.5709		
Glu	177	-13.6533	-13.2409	1.42487	-1.83724
Tyr	245	-13.181	-13.181		
Asp	246	-11.3111	-11.1518	1.17631	-1.3356
Trp	384	-10.8192	-10.8192		
Met	243	-4.83062	-4.83062		
Asp	175	-4.20281	-3.84319	0.83266	-1.19229
Phe	76	-2.0928	-2.0928		
Tyr	178	-1.22348	-1.22348		
Arg	301	-0.731935	-0.696286	-0.03565	
Gly	136	-0.408465	-0.408465		

Table S45. Amino acid residues contribution to **7** in AfChiB1.

Residue	ID	Total	EPair	EElec (r > 4.5)	EElec (r < 4.5)
Asp	246	-26.6469	-23.9636	-1.39038	-1.29288
Trp	137	-16.4748	-16.4748		
Trp	384	-15.5906	-15.5906		
Glu	177	-15.2386	-10.9332	-1.93999	-2.36543
Tyr	247	-11.0955	-11.0955		
Phe	273	-10.9115	-10.9115		
Tyr	245	-10.5104	-10.5104		
Tyr	178	-10.4277	-10.4277		
Met	243	-9.38134	-9.38134		
Ala	220	-6.18543	-6.18543		
Phe	251	-6.13878	-6.13878		
Phe	76	-2.87919	-2.87919		
Gly	221	-2.30278	-2.30278		
Gly	136	-2.29873	-2.29873		
Pro	219	-2.24794	-2.24794		
Lys	224	-1.37386	-1.32875	-0.0451065	
Tyr	48	-1.06001	-1.06001		
Glu	322	-0.931856	0	-0.931856	
Asp	385	-0.902851	0	-0.902851	
Asp	173	-0.82098	0	-0.82098	
Ala	248	-0.401551	-0.401551		
Ala	217	-0.303865	-0.303865		
Arg	57	0.331846	0	0.331846	

Lys	369	0.40519	0	0.40519	
Arg	301	0.490135	0	0.490135	

Table S46. Amino acid residues contribution to **8** in AfChiB1.

Residue	ID	Total	EPair	EElec (r > 4.5)	EElec (r < 4.5)
Asp	246	-23.5734	-21.198	-2.14784	-0.227577
Met	243	-15.9	-15.9		
Tyr	245	-15.725	-15.725		
Trp	384	-12.2138	-12.2138		
Trp	137	-11.4885	-11.4885		
Glu	177	-9.00304	-7.47583	-1.47912	-0.0480973
Tyr	178	-5.79061	-5.79061		
Tyr	247	-5.46925	-5.46925		
Tyr	48	-5.24261	-5.24261		
Phe	76	-5.22875	-5.22875		
Phe	251	-4.61487	-4.61487		
Phe	273	-3.53128	-3.53128		
Gly	136	-1.58066	-1.58066		
Lys	224	-1.27584	-2.04321	0.693146	0.0742283
Gly	221	-1.24643	-1.24643		
Asp	173	-1.01986	0	-1.01986	
Asp	385	-1.00436	0	-1.00436	
Glu	322	-0.854423	0	-0.854423	
Ala	217	-0.817627	-0.817627		
Ala	248	-0.58401	-0.58401		
Tyr	299	-0.43768	-0.43768		
Ser	252	-0.372601	-0.372601		
Ala	220	-0.315677	-0.315677		
Arg	57	0.336843	0	0.336843	
Lys	369	0.367628	0	0.367628	
Arg	301	1.06002	0	1.06002	
Asp	175	2.30133	6.12296	-1.85074	-1.97089

Table S47. Energy interaction values (kcal/mol) of the final candidates from flexible docking with AChiB1 and CHIT1. Candidates' aLogP values are also displayed.

Ligand	MolDock score (AfChiB1)	MolDock Score (CHIT1)	aLogP
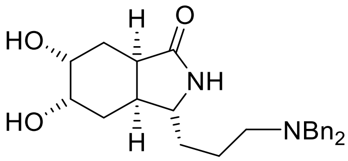 **1**	-161.06	-172.91	2.28
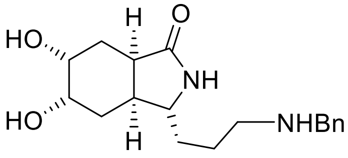 **2**	-140.51	-131.316	1.11
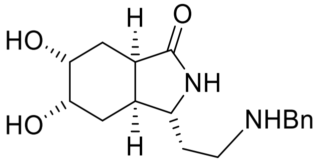 **3**	-117.14	-141.49	0.25
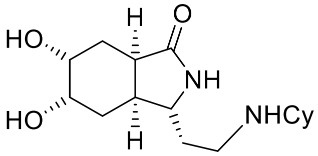 **4**	-135.19	-145.50	0.66
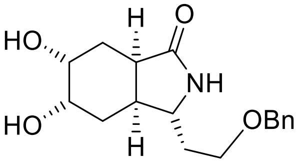 **5**	-135.851	Pose not found	0.39

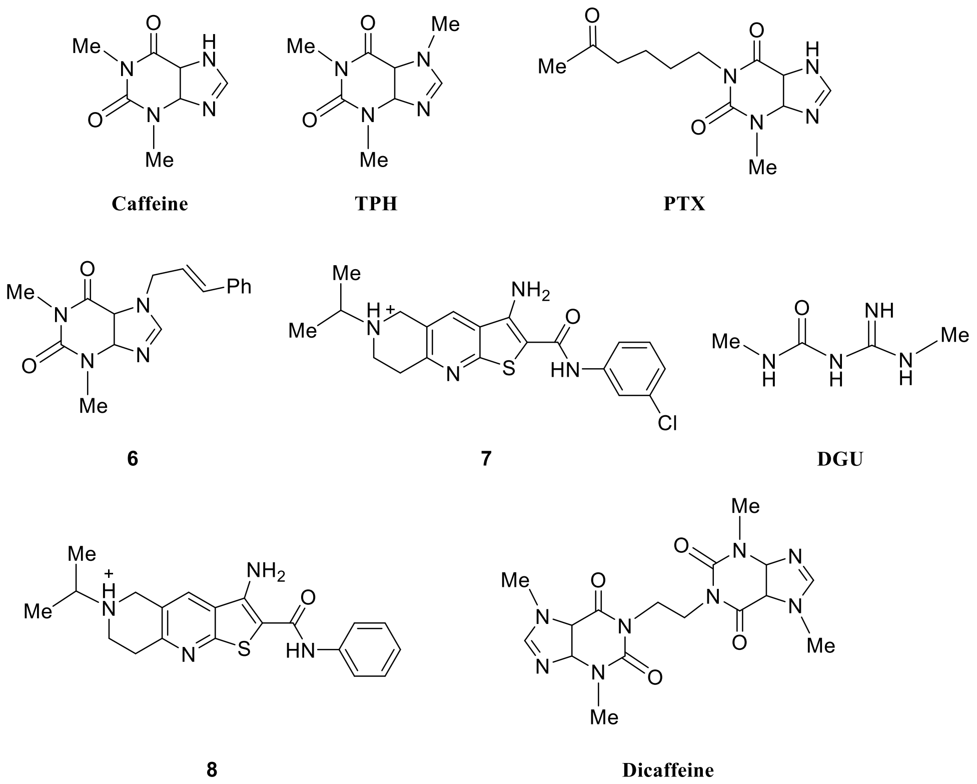

Figure S4. Structures used for model construction: Caffeine (PDB: 2A3B), Pentoxifylline (PTX; PDB: 2A3C), DGU (PDB: 3CH9), inhibitors **6** (Schüttelkopf et al., 2006), **7** (Jiang et al., 2016), **8** (Jiang et al., 2016), and Theophylline (TPH; PDB: 2A3A)

Table S48. Statistical parameters from model construction.

No.Var.	Q2loo	R2	R2adj	Kx	Kxy	s		
2	78.88	92.09	88.93	57.37	56.59	0.2606		
Object	Y Exp.	Y-Calc	Y-Pred	Hat	Err.Calc.	Err.Pred.	Std.Err.Calc.	Std.Err.Pred.
6	2.72	2.43	2.34	0.223	-0.29	-0.37	-1.26	-1.63
Caffeine	2.67	2.85	2.94	0.314	0.18	0.27	0.84	1.23
DGU	2.7	2.97	3.09	0.297	0.27	0.39	1.25	1.78
Dicaffeine	0.68	0.78	1.19	0.813 *	0.09	0.51	0.84	4.48
PTX	2.1	1.84	1.77	0.229	-0.26	-0.33	-1.13	-1.46
THP	3.18	2.96	2.86	0.301	-0.22	-0.31	-1	-1.44
7	1.71	1.84	1.96	0.504	0.12	0.25	0.67	1.35
8	1.97	2.06	2.11	0.319	0.09	0.14	0.44	0.64

Table S49. Estimation of predicted IC_{50} from equation 1 for ligand **1-5**.

**Ligand**	**Predicted IC_{50}** (μM)
1	62.78
2	97.36
3	190.86
4	103.25
5	90.86

Table S50. Swiss ADME values for each ligand **1-5**

**Ligand**	**1**	**2**	**3**	**4**	**5**
MW	408.53	318.41	304.38	296.41	305.37
#Heavy atoms	30	23	22	21	22
#Aromatic heavy atoms	12	6	6	0	6
Fraction Csp3	0.48	0.61	0.59	0.94	0.59
#Rotatable bonds	8	6	5	4	5
#H-bond acceptors	4	4	4	4	4
#H-bond donors	3	4	4	4	3
MR	121.49	92.1	87.29	84.73	85.58
TPSA	72.8	81.59	81.59	81.59	78.79
iLOGP	3.36	2.07	2.17	2.31	1.99
XLOGP3	2.6	0.64	0.28	0.6	0.56
WLOGP	2.03	0.27	-0.12	0.16	0.31
MLOGP	2.31	1.02	0.78	0.83	0.78
Silicos-IT Log P	2.98	1.54	1.16	0.89	1.48
Consensus Log P	2.65	1.11	0.86	0.96	1.02
ESOL Log S	-3.78	-2.01	-1.78	-1.79	-1.96
ESOL Solubility (mg/ml)	6.80E-02	3.08E+00	5.11E+00	4.79E+00	3.36E+00
ESOL Solubility (mol/l)	1.66E-04	9.67E-03	1.68E-02	1.62E-02	1.10E-02
ESOL Class	Soluble	Soluble	Very soluble	Very soluble	Very soluble
Ali Log S	-3.78	-1.93	-1.56	-1.89	-1.79
Ali Solubility (mg/ml)	6.81E-02	3.75E+00	8.48E+00	3.84E+00	4.99E+00
Ali Solubility (mol/l)	1.67E-04	1.18E-02	2.79E-02	1.30E-02	1.63E-02
Ali Class	Soluble	Very soluble	Very soluble	Very soluble	Very soluble
Silicos-IT LogSw	-6.04	-3.9	-3.51	-2.02	-3.19
Silicos-IT Solubility (mg/ml)	3.68E-04	3.97E-02	9.49E-02	2.80E+00	1.95E-01
Silicos-IT Solubility (mol/l)	9.02E-07	1.25E-04	3.12E-04	9.44E-03	6.38E-04

Silicos-IT class	Poorly soluble	Soluble	Soluble	Soluble	Soluble
GI absorption	High	High	High	High	High
BBB permeant	Yes	No	No	No	No
Pgp substrate	Yes	Yes	Yes	Yes	Yes
CYP1A2 inhibitor	No	No	No	No	No
CYP2C19 inhibitor	No	No	No	No	No
CYP2C9 inhibitor	No	No	No	No	No
CYP2D6 inhibitor	Yes	No	No	No	No
CYP3A4 inhibitor	No	No	No	No	No
log Kp (cm/s)	-6.95	-7.79	-7.96	-7.68	-7.77
Lipinski #violations	0	0	0	0	0
Ghose #violations	0	0	0	0	0
Veber #violations	0	0	0	0	0
Egan #violations	0	0	0	0	0
Muegge #violations	0	0	0	0	0
Bioavailability Score	0.55	0.55	0.55	0.55	0.55
PAINS #alerts	0	0	0	0	0
Brenk #alerts	0	0	0	0	0
Leadlikeness #violations	2	0	0	0	0
Synthetic Accessibility	3.93	3.41	3.34	3.74	3.67
